# Biopsychosocial factors of gaming disorder: a systematic review employing screening tools with well-defined psychometric properties

**DOI:** 10.3389/fpsyt.2023.1200230

**Published:** 2023-07-18

**Authors:** Rose Seoyoung Chang, Minju Lee, Jooyeon Jamie Im, Kee-Hong Choi, Jueun Kim, Jeanyung Chey, Suk-Ho Shin, Woo-Young Ahn

**Affiliations:** ^1^Department of Psychology, Seoul National University, Seoul, Republic of Korea; ^2^School of Psychology, Korea University, Seoul, Republic of Korea; ^3^Department of Psychology, Chungnam National University, Daejeon, Republic of Korea; ^4^Department of Child and Adolescent Psychiatry, Dr. Shin’s Neuropsychiatric Clinic, Seoul, Republic of Korea

**Keywords:** gaming disorder, GD, problematic gaming, pathological gaming, behavioral addiction, biopsychosocial model

## Abstract

**Background and aims:**

Considering the growing number of gamers worldwide and increasing public concerns regarding the negative consequences of problematic gaming, the aim of the present systematic review was to provide a comprehensive overview of gaming disorder (GD) by identifying empirical studies that investigate biological, psychological, and social factors of GD using screening tools with well-defined psychometric properties.

**Materials and methods:**

A systematic literature search was conducted through PsycINFO, PubMed, RISS, and KISS, and papers published up to January 2022 were included. Studies were screened based on the GD diagnostic tool usage, and only five scales with well-established psychometric properties were included. A total of 93 studies were included in the synthesis, and the results were classified into three groups based on biological, psychological, and social factors.

**Results:**

Biological factors (*n* = 8) included reward, self-concept, brain structure, and functional connectivity. Psychological factors (*n* = 67) included psychiatric symptoms, psychological health, emotion regulation, personality traits, and other dimensions. Social factors (*n* = 29) included family, social interaction, culture, school, and social support.

**Discussion:**

When the excess amount of assessment tools with varying psychometric properties were controlled for, mixed results were observed with regards to impulsivity, social relations, and family-related factors, and some domains suffered from a lack of study results to confirm any relevant patterns.

**Conclusion:**

More longitudinal and neurobiological studies, consensus on a diagnostic tool with well-defined psychometric properties, and an in-depth understanding of gaming-related factors should be established to settle the debate regarding psychometric weaknesses of the current diagnostic system and for GD to gain greater legitimacy in the field of behavioral addiction.

## 1. Introduction

Gaming is a widely and commonly enjoyed leisure activity. The number of active video gamers worldwide has marked 2.69 billion by the end of 2020 and is expected to continue its growing pattern (1). The recent trend towards more gaming engagement has been partly attributed to the widespread COVID-19 lockdown, which has hindered engagement in other interpersonal connections (2). While healthy usage of gaming brings certain emotional, social, and educational benefits, problematic gaming has been associated with negative consequences (3–6). Taking the inevitable link between the perils of addiction to games and mental health into account, Internet gaming disorder was included in the 5th edition of the *Diagnostic and Statistical Manual of Mental Disorders* (DSM-5) as a “condition for further study” (7). Furthermore, gaming disorder (GD) was recently included in the 11th Revision of the International Classification of Diseases [ICD-11; (8)].

However, there has been an ongoing debate among experts and researchers in the field on the issue of GD being officially recognized as a non-substance addiction disorder (9, 10). One of the main difficulties in settling this debate stems from the implementation of different GD screening and assessment tools with varying psychometric properties. King et al. (11) reported that more than 40 diagnostic tools with different evaluative properties were being employed in GD research studies. The number of screening tools continues to grow due to the adaption or development of new tools in lieu of utilizing already established ones. The plethora of new tools with questionable psychometric properties led many researchers in the field to criticize assessment and measure inconsistencies in GD papers and to argue that adequate psychometric properties of the scales need to be established for the effective comparability of the study results (12–14). Therefore, in order to systematically review GD papers effectively, it is crucial to start from similar, if not the same, criteria for GD as much as possible. In other words, the excessive tool usage in GD research needs to be controlled in an attempt to accurately compare and analyze the results of existing GD studies.

King et al. (11) evaluated all available GD screening tools according to their DSM/ICD coverage, empirical evidence, and psychometric properties. While no single tool was found to be superior, they reported five scales with greater evidential support for their psychometric properties: (1) Assessment of Internet and Computer Addiction Scale-Gaming (AICA-Sgaming), (2) Seven-Item Game Addiction Scale (GAS-7), (3) Ten-Item Internet Gaming Disorder Test (IGDT-10), (4) Internet Gaming Disorder Scale-Short Form (IGDS9-SF), and (5) Internet Gaming Disorder Scale (Lemmens IGD-9). In a recent meta-analysis, all five instruments were found to have good internal consistency and test–retest reliability (15). Thus, in order to control for the abundance of GD tools in the field, the present review sought to implement an incisive approach of gathering and comparing results of studies that have utilized one of these five GD tools with relatively greater evidential support.

Another difficulty in settling the debate on the issue of GD being officially recognized as an addictive disorder stems from the lack of systematic reviews of scientific literature on GD that identify comprehensive factors associated with gaming. To our knowledge, there exist a small number of comprehensive systematic reviews. Mihara and Higuchi (16) reviewed cross-sectional and longitudinal epidemiological studies of GD that were published up to May 2016. They reported that the comparison of the findings was hindered by insufficient longitudinal studies along with diversified methodologies utilized in each study. Paulus et al. (17) reviewed literature that investigated GD factors in children and adolescents that were published up to August 2016. They concluded that while GD can be characterized as a complex and endangering disorder, its concept and pathways leading to it cannot be fully analyzed due to the lack of longitudinal studies. Similarly, Sugaya et al. (18) reviewed literature that investigated biopsychosocial factors of GD in children and adolescents that were published up to February 2018. They summarized various factors that were associated with the presence of GD yet acknowledged that diverse methods of classifications yielded differences in results.

None of the prior reviews have imposed restrictions on the diagnostic tool usage, which hindered the comparison of the findings. Furthermore, there is a need for an updated literature search considering the increased attention GD has received since the ICD-11 release year as well as the rapid growth of novel coronavirus disease 19 (COVID-19). Therefore, the aim of the present review was to overcome the limitations of existing reviews and to provide a more up-to-date, comprehensive overview of GD by systematically identifying and summarizing the findings of studies that used one of five aforementioned diagnostic tools to investigate biopsychosocial factors of GD. To clarify, the purpose of the present review is not to evaluate the best GD diagnostic tools that must be used in investigations but rather to analyze GD patterns, if they exist, after controlling for various diagnostic tools used in articles.

## 2. Materials and methods

The present systematic review sought to collect the findings of all published studies reporting biological, psychological, or social factors related to gaming disorder. Included literature used various terms for describing problematic gaming behaviors; to maintain consistency and to avoid confusion, we use the term GD for all classification styles. A systematic approach following the PRISMA (Preferred Reporting Items for Systematic Reviews and Meta-Analyses) guidelines were utilized (19).

### 2.1. Search process and eligibility criteria

**Figure 1:**
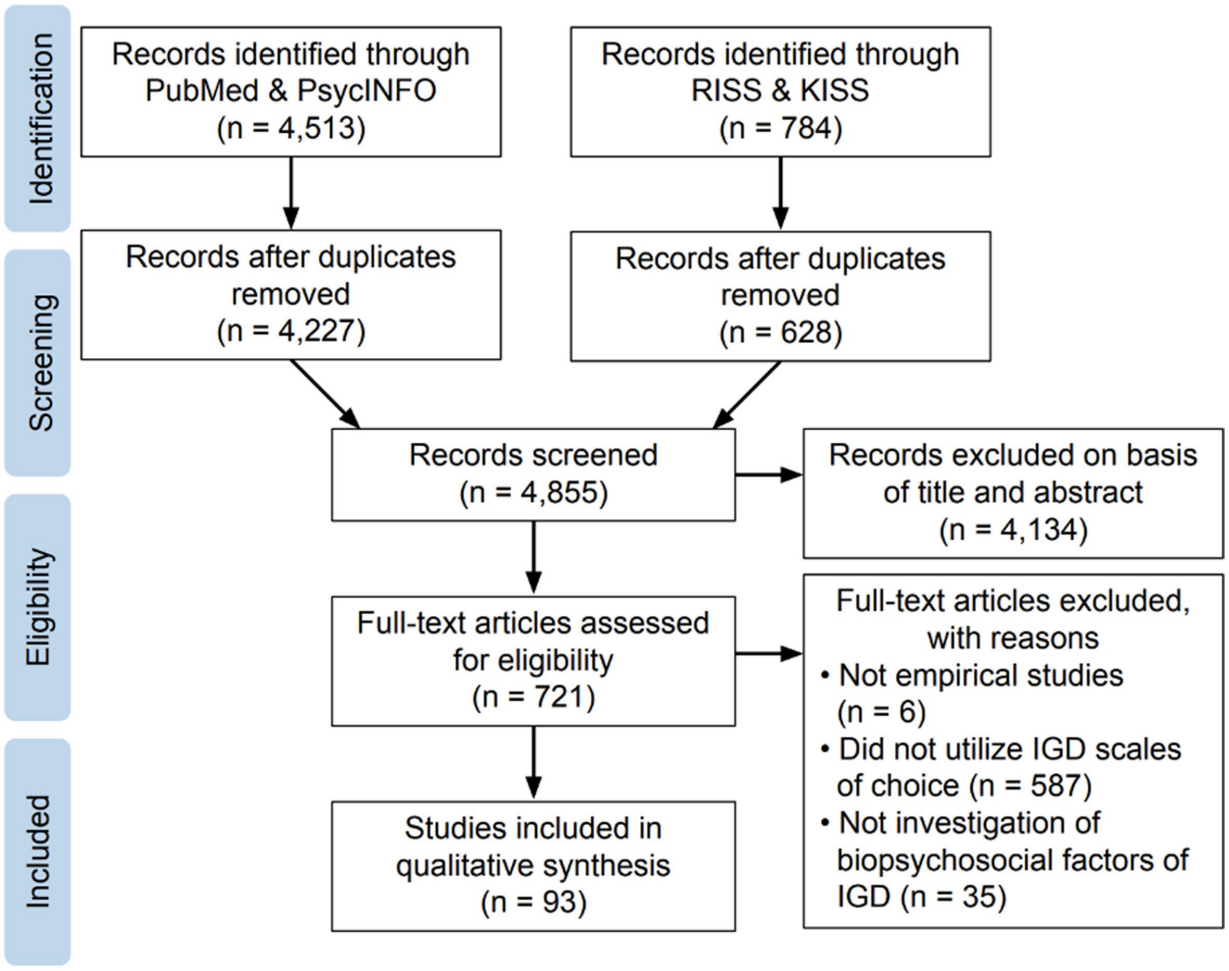
Flow diagram of the paper selection process for the systematic review.

Figure 1 presents a summary of the search process for the systematic review. The search included all publication years (up to January 2022) using four large electronic databases: PsycINFO, PubMed, RISS (Research Information Sharing Service; http://www.riss.kr/index.do), and KISS (Koreanstudies Information Service System; https://kiss.kstudy.com/index.asp). The latter two are large South Korean research databases and were implemented in the search for the inclusion of relevant literature published in Korean; this step was added, considering the high internet penetration rate in Asia and the importance of exploring international databases (20). The title or abstract terms used for the search for PsycINFO and PubMed were: (“pathology*” OR “problem*” OR “compulsive” OR “overuse” OR “abuse” OR “dependen*” OR “disorder*” OR “excess*” OR “addict*”) AND (“video” OR “computer” OR “internet” OR “online” OR “offline”) AND (“gaming” OR “game”). The terms used for the search for RISS and KISS were: “gaming addiction” OR “gaming disorder” OR “excessive gaming” OR “gaming use disorder” OR “gaming dependence” translated in Korean. The discrepancy in search terms was due to language and search setting differences. The initial search yielded 5,297 results. After removing the duplicates, the titles and abstracts of the remaining results (*n* = 4,855) were evaluated according to the following inclusion criteria: (1) published in peer-reviewed journals, (2) written in English or Korean, (3) empirical studies with primary data, (4) full-text availability, (5) investigated biopsychosocial characteristics of GD, and (6) utilized one of five scales (GAS-7, AICA-Sgaming, IGDS9-SF, Lemmens IGD-9, or IGDT10) to assess GD symptoms. The remaining full-text articles (*n* = 721) were read thoroughly. Six studies were not empirical studies, 587 studies did not utilize GD scales of choice, and 35 studies did not investigate biopsychosocial factors of GD thus were excluded. This resulted in 93 articles being included in the qualitative synthesis. The quality of each article was evaluated using the Kmet quality checklist [(21); see Appendix A in Supplementary material]. Throughout the search process, three researchers independently selected articles that met the inclusion criteria and any disagreements were resolved by consensus.

### 2.2. Scale descriptions

Studies were selected based on the GD screening tools recommended by King et al. (11): IGDS9-SF, GAS-7, Lemmens IGD-9, AICA-Sgaming, and IGDT-10. These were chosen upon a critical evaluation of all available GD tools in terms of their DSM/ICD coverage, quantitative evidence base, and psychometric properties. See Appendix B in the Supplementary material for the detailed descriptions for each scale.

### 2.3. Data extraction and synthesis

Information from 93 studies was extracted regarding the participant characteristics, GD diagnostic information, key findings, and statistical analyses used. Results were then grouped by categories (biological, psychological, or social) and corresponding subcategories.

## 3. Results

### 3.1. Study characteristics

Information extracted from the studies included in the review are presented in Tables 1–3. Results were classified into three groups: biological (*n* = 8), psychological (*n* = 67), or social (*n* = 29). Since psychological and social factors were often studied together, 11 studies were included in both groups. Approximately 70% of the studies were published within the past 4 years (2019–2022). Geographically, studies were carried out in Europe (*n* = 40), Asia (*n* = 38 including *n* = 13 from the Middle East), North America (*n* = 3), South America (*n* = 2), and worldwide (*n* = 10). As for the gender of study samples, six studies reported samples of only males while the remaining reported samples of all genders. The study samples included young adults/adult population (*n* = 51), adolescents/children (*n* = 31), or all age ranges (*n* = 11). Following the inclusion criteria, all studies utilized one of the five diagnostic scales: IGDS9-SF (*n* = 40), GAS-7 (*n* = 23), Lemmens IGD-9 (*n* = 17), AICA-Sgaming (*n* = 7), and IGDT10 (*n* = 6). When assessed for quality, the summary score (total score divided by the total possible score) of all studies ranged from 0.75 to 1.00, which met the conservative cut-point (0.75) suggested by the quality assessment guidelines (21).

**Table 1:** Biological findings in gaming disorder (IGD) included.

Author(s) (year)	Participants (country of study)	Diagnostic scale for IGD	Measures	Main findings	Design
<Reward>					
Ariatama et al. (22)	N = 48, 20 ~ 40 years (Indonesia)	IGDS9-SF	DAT level	IGD was associated with lower DAT level.	Cross-sectional
Duven et al. (23)	* males only PCG: N = 14, 24.29 ± 5.84 years. CG: N = 13, 23.31 ± 3.01 years (Germany)	AICA-Sgaming	functional EEG	PCG showed attenuated amplitude of P300 in response to rewards compared to CG. PCG showed prolonged latency and increased amplitude of N100 compared to CG.	Cross-sectional
Turel et al. (24)	intensive players: N = 26, 20.46 ± 2.10 years. HC: N = 26, 20.69 ± 2.21 years (China)	IGDS9-SF	fMRI	Players showed stronger activation in the ventral striatum compared to HC. Players showed weaker activation in the DLPFC compared to HC. Players showed higher activation in the left insula compared to HC when shown game-relating videos/cues.	Cross-sectional
<Self-concept>					
Dieter et al. (25)	Addicted: N = 15, 28.73 ± 7.73 years. Non-addicted: N = 17, 24.94 ± 4.16 years (Germany)	AICA-Sgaming	fMRI	Addicted group showed enhanced activation in the left AG compared to the non-addicted during avatar-related vs. self-related reflection. Addicted group showed stronger identification with the avatar in the game compared to the non-addicted.	Cross-sectional
Leménager et al. (26)	PG: N = 19, 25.68 ± 6.69 years. HC: N = 19, 27.68 ± 7.95 years. (Germany)	AICA-Sgaming	fMRI	PG showed hyperactivation in the left AG compared to HC during avatar reflection. PG considered their avatar’s popularity significantly superior than their own.	Cross-sectional
<Brain Structure>					
Choi et al. (27)	* males only IGD: N = 27, 29.45 ± 4.74 years. IGC: N = 29, 30.00 ± 5.75 years. NGC: N = 26, 27.21 ± 4.88 years (South Korea)	Lemmens IGD-9	sMRI	IGD showed decreased gray matter density in the left DLPFC compared to IGC and NGC.	Cross-sectional
< Functional Connectivity (FC) >					
Chun et al. (28)	IGD: N = 45, 27.76 ± 5.31 years. HC: N = 45, 25.29 ± 4.07 years (South Korea)	Lemmens IGD-9	rs-fMRI	IGD showed lower FC between the VLPFC and DLPFC and between the PPC and DLPFC in the CEN compared to HC.	Cross-sectional
Kim et al. (29)	* males only IGD: N = 22, 28.27 ± 5.33 years. HC: N = 24, 28.17 ± 5.93 years (South Korea)	Lemmens IGD-9	rs-fMRI	IGD showed lower FC from the bilateral OFC to frontal, striatal, temporal, and occipital regions compared to HC.	Cross-sectional

AG = angular gyrus, ANCOVA = analysis of covariance, ANOVA = analysis of variance, CEN = central executive network, CG = casual computer gaming, DAT = dopamine transporter, DLPFC = dorsolateral prefrontal cortex, EEG = electroencephalography, fMRI = functional magnetic resonance imaging, HC = healthy controls, IGC = internet gaming control, IGD = Internet gaming disorder, NGC = non-gaming control, OFC = orbitofrontal cortex, PCG = pathological computer gaming, rs-fMRI = resting state-functional magnetic resonance imaging, sMRI = structural magnetic resonance imaging, VLPFC = ventrolateral prefrontal cortex.

**Table 2:** Psychological findings in gaming disorder (IGD) included.

Author(s) (year)	Participants (country of study)	Diagnostic criteria	Measures	Psychological factors	Analytical strategy
<Psychiatric symptoms>					
Evren et al. (30, 31)	Probable ADHD Absent: N = 355, 24.48 ± 5.44 years. Present: N = 102, 23.43 ± 3.63 years (Turkey)	Lemmens IGD-9	ASRS-v1.1	IGD was associated with higher probable ADHD symptoms.	Cross-sectional
Bonnaire and Baptista (32)	PG: N = 273, 20.5 ± 2.5 years. NPG: N = 156, 21.2 ± 2.6 years (France)	GAS-7	TAS-20 HADS	IGD was associated with being alexithymic, depression scores, and anxiety scores.	Cross-sectional
* De Pasquale et al. (33)	N = 566, 22.74 ± 4.83 years (Italy)	IGDS9-SF	SCL-90-R The APA symptom checklist	IGD was associated with higher symptoms of somatization, depression, and sleep disturbances.	Cross-sectional
Concerto et al. (34)	N = 4,260, 18–55 years. (Italy)	IGDS9-SF	AQ ASRS	IGD was associated with autistic traits and ADHD symptoms after controlling for demographic variables.	Cross-sectional
* Haghbin et al. (35)	N = 326, high school students (Iran)	GAS-7	The Self-Control Scale ASRS-v1.1	The presence of ADHD was significantly related in the relationship between video game addiction and other variables (self-control and academic achievement).	Cross-sectional
Pontes (36)	N = 509, 13.02 ± 1.64 years (Portugal)	IGDS9-SF	The Bergen Facebook Addiction Scale DASS-21	IGD was associated with younger age, male gender, and SNS addiction. IGD affected depression, anxiety, and stress levels.	Cross-sectional
Horváth et al. (37)	N = 2,768, 16.73 ± 1.21 years (Hungary)	IGDT-10	Alcohol Consumption Illicit Drug Use	Polysubstance users presented higher levels of IGD symptoms compared to high-risk alcohol, moderate alcohol, and infrequent substance users.	Cross-sectional
Ismail et al. (38)	N = 237, 19 ~ 27 years (Malaysia)	IGDS9-SF	DASS-21	There was no association between IGD and anxiety symptoms.	Cross-sectional
Kircaburun et al. (39)	N = 242, 18.87 ± 4.57 years (Turkey)	IGDT10	Short depression-happiness scale CTQ Single item self-esteem scale Social anxiety scale for adolescents short form ULS-4	Depressive symptoms fully mediated the relationship between childhood trauma and IGD. IGD was associated with higher levels of emotional trauma, loneliness, self-esteem, and social anxiety.	Cross-sectional
* Wartberg et al. (40)	N = 1,531, 18.86 ± 4.06 years (Germany)	Lemmens IGD-9	Patient Health Questionnaire-2 The Generalized Anxiety Disorder Scale-2	IGD was associated with higher levels of depression and anxiety. IGD was associated with more frequent neglect of social contacts.	Cross-sectional
* Männikkö et al. (41)	N = 293, 18.7 ± 3.4 years (Finland)	GAS-7	Social health questionnaire SWLS	IGD was associated with higher depression, fatigue, sleep problems, concentration problems, and anxiety. Depression and a preference for online social interaction predicted IGD.	Cross-sectional
Mentzoni et al. (42)	N = 2,500, 15 ~ 40 years (Norway)	GASA (GAS-7)	SWLS HADS	IGD was associated with more antisocial behavior, anger control problems, emotional distress, and hyperactivity and inattention in adolescents.	Cross-sectional
Murray et al. (43)	ASD: N = 230, 31.32 ± 11.03 years. HC: N = 272, 29.51 ± 13.53 years (Ireland)	IGDT-10	AQ	Individuals in the ASD group showed higher IGD symptoms.	Cross-sectional
Musetti et al. (44)	N = 142/86/76/62 (passionate/occasional/preoccupied/disordered gamers), 21.64 ± 3.77 years (Italy)	IGDS9-SF	The Personality Inventory for DSM-5 Brief Form	Disordered gamers presented highest levels of psychoticism, psychotic symptoms, and suicidal ideation compared to other groups.	Cross-sectional
Severo et al. (45)	N = 555, 14+ years. (Brazil)	IGDS9-SF	BDI PSQI The Mini-Social Phobia Inventory	IGD was associated with severe depressive symptoms. IGD was associated with poor sleep quality.	Cross-sectional
Singh et al. (46)	N = 306, 22.73 ± 3.97 years (India)	IGDS9-SF	Patient Health Questionnaire-9	IGD was associated with higher levels of depressive symptoms.	Cross-sectional
Siste et al. (47)	N = 639, 20.23 ± 0.13 years (Indonesia)	IGDT-10	SCL-90-R The Indonesian version of the modified Temperament and Character Inventory	IGD was associated with more experiences of all psychopathologies assessed, excepting phobic anxiety.	Cross-sectional
Stavropoulos et al. (48)	Australian: N = 164, 23.01 ± 3.35 years. American: N = 457, 25.25 ± 2.76 years (Australia and USA)	IGDS9-SF	ASRS-v1.1 EPQR-A	Those presenting higher inattention and hyperactivity symptoms exhibited more IGD symptoms.	Cross-sectional
Stavropoulos et al. (49)	N = 964, 25.74 years (Australia and USA)	IGDS9-SF	DASS-21	IGD was associated with more depression symptoms.	Cross-sectional
* Stockdale and Coyne (50)	VGA: N = 87, 20.80 ± 2.18 years. HC: N = 87, 20.80 ± 2.18 years (USA)	Lemmens IGD-9	ASRS-v1.1 The Neuro-QOL BPAQ-SF The Cyber Pornography Use Inventory-9	IGD was associated with higher degree of ADHD symptoms, anxiety, depression, aggression, and pornography addiction.	Cross-sectional
Turhan-Gürbüz et al. (51)	N = 93, 15–24 years (Turkey)	IGDS9-SF	Substance use disorder	Lower IGD scores were found in the substance use patient group compared to the non-patient group.	Cross-sectional
Vally (52)	N = 214, 20.64 ± 4.34 years (UAE)	IGDS9-SF	ASRS-v1.1	Increased symptoms of inattention and impulsivity were associated with elevated risk for IGD.	Cross-sectional
Choi (53)	* males only N = 240, 20.53 ± 1.39 years (China)	IGDS9-SF	State–Trait Anxiety Inventory SWLS	IGD was associated with lower levels of life satisfaction. Higher levels of anxiety fully mediated the relationship between IGD and life satisfaction.	Cross-sectional
Wong et al. (54)	N = 300, 20.89 ± 1.48 years (Hong Kong)	IGDS9-SF	BSMAS DASS-21 PSQI	IGD was associated with higher levels of social media addiction, depression, anxiety, and stress. IGD was associated with poor sleep quality.	Cross-sectional
<Psychological health>					
Buiza-Aguado et al. (55)	N = 708, 15.6 ± 2.7 years (Spain)	Lemmens IGD-9	The Self-Esteem Scale SWLS SDQ BPAQ ULS	IGD was negatively associated with self-esteem, life satisfaction, and prosocial behaviors. IGD was positively associated with loneliness and physical aggression.	Cross-sectional
Kim et al. (56)	Male: N = 209 Female: N = 429 Univ. students (South Korea)	Lemmens IGD-9	Suicidal Probability Scale The event related rumination inventory	Males have shown higher tendency to IGD. Females have shown higher intrusive rumination and suicide risk.	Cross-sectional
Evren et al. (57)	NSSI present: N = 207, 21.51 ± 3.14 years. NSSI absent: N = 803, 21.93 ± 3.44 years (Turkey)	IGDS9-SF	Lifetime history of NSSI	IGD was associated with the presence of lifetime NSSI behaviors.	Cross-sectional
Wartberg et al. (58)	T1 = 1,095, T2 = 985 dyads 12.98 ± 0.82 years (Germany)	Lemmens IGD-9	The Young Diagnostic Questionnaire The Reynolds Adolescent Adjustment Screening Inventory The Strengths and Difficulties Questionnaire The Generalized Anxiety Disorder Scale-2	IGD at T2 was predicted by more self-esteem problems at T1. IGD at T2 was predicted by higher symptoms of hyperactivity/inattention at T1.	Longitudinal
* Wartberg et al. (59)	N = 1,095, 12.99 ± 0.82 years (Germany)	Lemmens IGD-9	SPS-J-II SDQ	IGD was associated with higher levels of adolescent antisocial behavior, anger control problems, and hyperactivity and inattention.	Cross-sectional
Phan et al. (60)	IGD-: N = 182, 14.83 ± 0.05 years. IGD+: N = 516, 14.86 ± 0.09 years. Patients: N = 43, 15.74 ± 0.38 years (France)	GAS-7	Life Satisfaction Scale Adolescent Depression Rating Scale The Liebowitz Social Anxiety Scale	IGD was associated with decreased male’s quality of life, which was not found in females.	Cross-sectional
* Teng et al. (61)	T1 = 1,054, T2 = 924, T3 = 931 17–21 (China)	IGDS9-SF	Rosenberg Self-esteem Scale Multidimensional Scale of Perceived Social Support The Satisfaction with Life Scale	IGD negatively affected self-esteem, social support, and life satisfaction, but not vice versa.	Longitudinal
<Emotion regulation>					
Evren et al. (30, 31)	N = 987, 23.65 ± 6.37 years (Turkey)	IGDS9-SF	TAS-20 BPAQ SCL-90-R	Higher levels of alexithymia and aggression predicted IGD symptoms.	Cross-sectional
Wartberg et al. (62)	T1: N = 1,095, 12.99 ± 0.82 years. T2: N = 985, 13.89 ± 0.89 years (Germany)	Lemmens IGD-9	SPS-J-II	IGD at T1 was a predictor for more subsequent emotional distress at T2.	Longitudinal
Kim and Kwon (63)	IGD: N = 49, 26.00 ± 5.00 years. Control: N = 50, 24.26 ± 6.59 years (South Korea)	Lemmens IGD-9	SWLS Brief Symptom Inventory Korean version of the Difficulties in Emotion Regulation Scale Korean Version of the Self-Control Scale	IGD was associated with higher levels of negative emotions and lower levels of positive emotions.	Cross-sectional
Müller and Bonnaire (64)	PG = 37, NPG = 133, NG = 37 19.02 ± 4.20 years (France)	GAS-7	The Player-Avatar Identification Scale The Difficulties in Emotion Regulation Scale The Emotion Regulation Questionnaire for Children and Adolescents The Interpersonal Regulation Questionnaire	PG had higher scores in lack of emotional consciousness, lack of emotional clarity, and expressive suppression compared to NG and NPG. PG had lower scores in cognitive reappraisal and interpersonal emotion regulation compared to NG and NPG.	Cross-sectional
* T’ng et al. (65)	N = 410, 23.9 years (Malaysia)	IGDS9-SF	BPAQ	IGD was associated with more physical and verbal aggression, anger, and hostility.	Cross-sectional
* Uçur and Dönmez (66)	With PIG: N = 144 Without PIG: N = 923 14.80 ± 1.60 years (Turkey)	GAS-7	The Difficulties in emotion regulation scale	IGD was associated with high emotional dysregulation.	Cross-sectional
< Reward, discounting and impulsivity >					
Acuff et al. (67)	N = 1,406, 20.92 ± 3.71 years (Argentina, Australia, India, Malaysia, UK, and US)	GAS-7	The Problematic Internet Use Questionnaire Internet Purchase Task The eight-item Delayed Reward Discounting Task	There was no significant association between reward sensitivity and online gaming.	Cross-sectional
Cerniglia et al. (68)	N = 656, 16.32 ± 1.54 years (Italy)	IGDS9-SF	• BIS-11-A • SCL-90-R	• IGD was associated with higher impulsivity levels.	• Cross-sectional
Wölfling et al. (69)	* males only IGD: N = 30, 26.9 ± 5.97 years. GD: N = 31, 35.0 ± 11.44 years. HC: N = 27, 25.6 ± 3.25 years (Germany)	AICA-Sgaming	The two-item Lie/Bet-Questionnaire BIS-11	There were no group differences in impulsivity levels IGD showed faster ability to adopt advantageous decision strategies.	Cross-sectional
Macur and Pontes (70)	N = 1,023, 13.44 ± 0.59 years (Slovenia)	IGDS9-SF	Self-control measure	High risk gamers showed lower levels of self-control compared to low risk gamers.	Cross-sectional
Moudiab and Spada (71)	N = 79, 21.3 ± 3.2 years (United Kingdom)	IGDT10	Motives for Online Gaming Questionnaire Maladaptive Gaming-Related Cognitions Scale	Higher maladaptive cognitions relating to overvaluing of gaming rewards and motives relating to coping and skills development predicted IGD.	Cross-sectional
<Personality traits>					
Borzikowsky and Bernhardt (72)	N = 305, 28.44 ± 8.88 years (Germany)	GAS-7	German Version of the Short Grit Scale	Grit significantly reduced the likelihood of IGD.	Cross-sectional
Müller et al. (73)	* males only N = 115/74/122/93 (IGD/Clinical control/GD/HC), 16 + years (Germany)	AICA-Sgaming	The NEO Five-Factor Inventory	IGD was positively associated with neuroticism. IGD was negatively associated with extraversion, openness, agreeableness, and conscientiousness.	Cross-sectional
Müller et al. (74)	IRD (Internet related disorder) = 102, GD (gambling) = 106, CG(HC) = 89 25.5 ± 8.11 years (Germany)	AICA-Sgaming	The Personality Inventory for DSM-5–Brief Form The Virtual Expectancy Questionnaire The Global Assessment of Functioning	IRD showed higher scores in each maladaptive trait compared to GD or HC.	Cross-sectional
Sánchez-Llorens et al. (75)	N = 119, 14.85 ± 0.79 years (Spain)	GAS-7	BFQ-NA	IGD was positively associated with neuroticism. IGD was negatively associated with extraversion and conscientiousness.	Cross-sectional
* Wittek et al. (76)	N = 3,389, 32.6 years. (addicted/problem/engaged/normal gamers; Norway)	GAS-7	The Mini International Item Pool An eight-item scale to assess psychosomatic health symptoms	IGD was positively associated with neuroticism. IGD was negatively associated with extraversion, agreeableness, and conscientiousness.	Cross-sectional
<Stress>					
* Andreetta et al. (77)	N = 605, 24.01 ± 6.11 years (Italy)	IGDS9-SF	Individualism and Collectivism Scale Cultural Orientation Scale DASS-21	Higher levels of stress increased IGD risk.	Cross-sectional
Canale et al. (78)	N = 605, 24.01 ± 6.11 years (Italy)	IGDS9-SF	PSS The 10-item Resilience Scale	IGD was associated with higher levels of perceived stress and lower psychological resilience.	Cross-sectional
Rajab et al. (79)	Addiction-Y: N = 130, 15.76 ± 1.71 years. Addiction-N: N = 2,407, 16.09 ± 1.58 years (Saudi Arabia)	GAS-7	PSS	IGD was associated with higher levels of stress.	Cross-sectional
<Cognitive approach>					
Bodi et al. (80)	Online gamer: N = 229, 28.34 ± 6.98 years. Offline: N = 217, 27.75 ± 6.93 years (France)	GAS-7	Video Game Cognition Scale HADS	Cognitive salience and completion predicted both online and offline IGD. Virtual comfort and time spent gaming predicted only online IGD.	Cross-sectional
Efrati, Kolubinski et al. (81)	N = 471, 15.73 ± 1.31 years (Israel)	IGDS9-SF	The Metacognitions Questionnaire 30 BIS-11 Food Thought Suppression Inventor	Thought suppression and impulsiveness mediated the Relationship between metacognitions and IGD.	Cross-sectional
<Self-concept>					
Concetta De Pasquale et al. (82)	N = 221, 21.56 ± 1.42 years (Italy)	IGDS9-SF	Dissociative Experience Scale for adolescents and young adults	IGD was positively associated with dissociative experiences (depersonalization and derealization; absorption and imaginative involvement; and passive influence).	Cross-sectional
Stavropoulos et al. (83)	N = 1,032, 24 ± 7 years (Australia and USA)	IGDS9-SF	The User-Avatar Questionnaire The Proteus-Effect Scale	IGD was higher in gamers who highly identified or fused with game avatars than those who differentiated themselves from the avatars.	Cross-sectional
<Sleep>					
Nakayama et al. (84)	PG = 35, NG = 514 12–13 (Japan)	IGDT10	Items regarding age, gender, night-time sleep, age at which weekly gaming started, time spent on Internet and gaming	IGD was associated with later bedtime and wake-up time.	Cross-sectional
Wang et al. (85)	N = 1,040, 20.32 ± 1.43 years (China)	GAS-7	SCL-90-R PSQI	Sleep quality mediated the relationship between IGD and psychological distress.	Cross-sectional
<Flow>					
Hu et al. (86)	N = 237, 18 ~ 59 years (mostly Australia and New Zealand)	IGDS9-SF	Online Flow Questionnaire	Flow fully mediated the relationship between preference for social games and IGD.	Cross-sectional
<Psychological Needs>					
Chamarro et al. (87)	N = 471, 21.73 ± 10.10 years (Spain)	IGDS9-SF	Need Satisfaction and Frustration Scale Internet Use Expectancies Scale	IGD was associated with higher level of need frustration. Game expectancies and time spent playing mediated this relationship.	Cross-sectional
<COVID-19>					
Chen et al. (88)	N = 1,357, 10.7 years. (China) Not overweight: N = 1,121, 10.67 ± 1.11 years. Overweight: N = 236, 10.78 ± 0.93 years.	IGDS9-SF	DASS-21 Smartphone Application-Based Addiction Scale BSMAS	IGD was associated with higher levels of stress, anxiety, and depression in non-overweight children. IGD was associated with lower levels of stress in overweight children.	Cross-sectional
Chen et al. (88)	N = 2,026, 10.71 ± 1.07 years (China)	IGDS9-SF	DASS-21 Smartphone Application-Based Addiction Scale BSMAS	IGD mediated the association between psychological distress (depression, anxiety, and stress) and increased gaming time during the school hiatus.	Cross-sectional
Fazeli et al. (89)	N = 1,512, 15.51 ± 2.75 years (Iran)	IGDS9-SF	DASS-21 Insomnia Severity Index Pediatric Quality of Life Inventory™ 4.0 Short Form	Depression, anxiety, and stress mediated the relationship between IGD and insomnia and quality of life during the pandemic.	Cross-sectional
Oka et al. (90)	N = 3,938, 46.6 ± 11.8 years (Japan)	Lemmens IGD-9	Demographic information COVID-19 infection status	IGD symptoms significantly increased during the pandemic. COVID-19 infection and young age were associated with IGD exacerbation.	Cross-sectional
Sallie et al. (91)	N = 1,344, 28.93 ± 12.46 years (International)	IGDS9-SF	COVID-19 related stress factors TIPI HADS SUPPS-P	Greater IGD severity during the quarantine was associated with greater depression, anxiety, and mood-based impulsivity.	Cross-sectional
Teng et al. (92)	N = 1,778 (China) Children: N = 875 (4th grade) Adolescents: N = 903 (7th grade)	IGDS9-SF	Perceived COVID-19 impacts Center for Epidemiologic Studies Depression Scale State–Trait Anxiety Inventory	Video game use increased for both children and adolescents, and IGD increased only for adolescents during the pandemic. IGD was associated with higher symptoms of depression and anxiety.	Cross-sectional
Ting and Essau (93)	N = 178, 22.56 ± 2.93 years (Malaysia)	GAS-7	SSRQ FCV-19S K6	Time spent on gaming had increased during the lockdown. IGD was associated with lower levels of self-regulation and higher levels of COVID-19 fear and psychological distress.	Cross-sectional
Zhu et al. (94)	N = 2,863, 12.6 ± 1.32 years (Hong Kong)	GAS-7	Multidimensional Scale of Perceived Social Support Parental Monitoring Scale Patient Health Questionnaire-9 Generalized Anxiety Disorder-7 scale	IGD was associated with higher levels of loneliness.	Cross-sectional

* Also included in another (social) category. ASRS = Adult ADHD Self-Report Scale, AQ = Autism-spectrum Quotient, ASD = Autism Spectrum Disorder, BDI = the Beck Depression Inventory, BFQ-NA = Big Five Personality Questionnaire for children and adolescents, Big five (N = neuroticism; E = extraversion; O = openness; A = agreeableness; C = conscientiousness), BIS-11-A = the Barratt Impulsiveness Scale for Adolescents, BPAQ-SF = the Short-Form Buss Perry Aggression Questionnaire, BSMAS = Bergen Social Media Addiction Scale, CTQ = Childhood Trauma Questionnaire, DASS-21 = Depression Anxiety Stress Scales, EPQR-A = Eysenck personality questionnaire revised abbreviated form, FCV-19S = Fear of COVID-19 Scale, GA = Gaming Addiction, GD = Gambling Disorder, HADS = the Hospital Anxiety and Depression Scale, K6 = Kessler Distress Scale, NSSI = Non-Suicidal Self-Injury, PG = Pathological Gaming, PSQI = Pittsburgh Sleep Quality Index, PSS = the Perceived Stress Scale, SCL-90-R = the Symptom Checklist-90-R, SDQ = the Strengths and Difficulties Questionnaire, SMA = Social Media Addiction, SPS-J-II = Screening psychischer Störungen im Jugendalter-II; German adaptation of the Reynolds Adolescent Adjustment Screening Inventory, SSRQ = Short Self-Regulation Questionnaire, SUPPS-P = UPPS-P Impulsive-Behavior Scale, SWLS = the Satisfaction with Life Scale, TAS-20 = Toronto Alexithymia Scale, TIPI = Ten-Item Personality Inventory, ULS = UCLA loneliness scale, VGA = Video Game Addiction.

**Table 3:** Social findings in gaming disorder (IGD) included.

Author(s) (year)	Participants (country of study)	Diagnostic scale for IGD	Measures	Social factors	Analysis
<Family>					
Bonnaire and Phan (95)	N = 434, 13.2 ± 0.5 years for males; 13.1 ± 0.5 years for females (France)	GAS-7	Parental attitudes to gaming use FRI	IGD was associated with banning video games, more rules about video game use, and poor family relationship.	Cross-sectional
Irmak and Erdogan (96)	N = 865, 16.5 ± 0.95 years (Turkey)	GAS-7	Family Environment Scale	Family environment predicted IGD in females only.	Cross-sectional
Koning et al. (97)	N = 354, 13.90 ± 0.74 years (Netherlands)	Lemmens IGD-9	Reactive restrictions Frequency of communication	Frequency of communication regarding Internet predicted IGD. IGD predicted reactive rules and lower quality of communication.	Cross-sectional
Lin et al. (98)	Participants: N = 320, 15.52 ± 1.98 years. Siblings: N = 320, 16.98 ± 2.91 years (Iran)	IGDS9-SF	DASS-21 ISI	Adolescents’ IGD scores affected their own depression, anxiety, stress, and insomnia. Adolescents’ siblings’ IGD scores affected adolescents’ depression anxiety, stress, and insomnia.	Cross-sectional
* Wartber et al. (40, 59, 99)	N = 1,095, 12.99 ± 0.82 years (Germany)	Lemmens IGD-9	PHQ-2 GAD-2	IGD was associated with higher parental anxiety and depression.	Cross-sectional
Stockdale and Coyne (100)	Mothers: N = 481, 30.97 ± 7.76 years. Fathers: N = 374, 32.44 ± 6.54 years. Children: 5.83 ± 3.50 months (USA)	Lemmens IGD-9	Parenting Sense of Competence Scale CES-10	IGD was associated with decreased feelings of parental efficacy. IGD was associated with increased depression for mothers and fathers.	Longitudinal
Sung et al. (101)	N = 546, college students (South Korea)	Lemmens IGD-9	ACE Event Related Rumination Inventory NEO-II	IGD tendency, rumination, and externalizing were higher in groups with adverse childhood experiences.	Cross-sectional
Throuvala et al. (102)	N = 172, 23.3 ± 1.83 years (Greece)	AICA-Sgaming	PARQ CSES	Perceived parental rejection influenced IGD only via the mediating factor of core self-evaluations.	Cross-sectional
* Teng et al. (103)	N = 1,054, 18.25 ± 0.73 years (China)	IGDS9-SF	IPPA	IGD predicted subsequent attachment with mother but negatively predicted father attachment. Father and mother attachment did not predict subsequent IGD.	Longitudinal
<Social interaction>					
* De Pasquale et al. (33)	N = 566, 22.74 ± 4.83 years (Italy)	IGDS9-SF	Social Adaptation Self Evaluation Scale	IGD was associated with poorer family and extra-family relationships.	Cross-sectional
Duman and Ozkara (104)	N = 318, all age range (Turkey)	GAS-7	FoMO scale Social Identity Scale	IGD was associated with a higher FoMO score. FoMO mediated the effect of social identity on IGD.	Cross-sectional
Festl et al. (105)	N = 4,207, 37.8 years (Germany)	GAS-7	California Psychological Inventory BSSS	IGD was associated with lower levels of sociability and less perceived social support.	Cross-sectional
* Wartberget al (40).	N = 1,531, 18.86 ± 4.06 years (Germany)	Lemmens IGD-9	How often do you neglect social contacts (e.g., friends or family members), who used to be important to you, because of computer game playing?	IGD was associated with more frequent neglect of social contacts.	Cross-sectional
* Männikkö et al. (41)	N = 293, 18.7 ± 3.4 years (Finland)	GAS-7	Preferences for online interaction scale	IGD was associated with lower sociability and a stronger preference for online interaction.	Cross-sectional
* T’ng et al. (65)	N = 410, 23.9 years (Malaysia)	IGDS9-SF	Revised UCLA Loneliness Scale	IGD was associated with greater loneliness.	Cross-sectional
Tullett-Prado et al. (106)	N = 1,032, 24 ± 7 years (USA, UK, Australia, New Zealand)	IGDS9-SF	Four Social Engagement questions	High IGD risk profile was linked with higher unemployment, lower levels of education, and living with divorced parents or friends.	Cross-sectional
Stavropoulos et al. (107)	N = 611, 23.38 ± 3.50 years for Australians; 25.25 ± 2.76 years for Americans (Australia and USA)	IGDS9-SF	HSWS	IGD was associated with higher Hikkikomori symptoms.	Cross-sectional
<Culture/Ethnicity>					
* Andreetta et al. (77)	N = 964, 25 ± 7 years (Australia, UK, and USA)	IGDS9-SF	ICS	Presence of vertically individualistic tendencies moderated the relationship between stress and IGD.	Cross-sectional
Stavropoulos et al. (83)	N = 1,032, 24 ± 7 years (Australia and USA)	IGDS9-SF	ICS	IGD was associated with a more vertically-individualistic cultural orientation. Cultural orientation moderated the relationship between inattention and IGD.	Cross-sectional
Stavropoulos et al. (49, 108)	N = 1,032 (worldwide)	IGDS9-SF	ICS	Those who are aversive to collectivism displayed higher IGD behaviors.	Cross-sectional
* Wittek et al. (76)	N = 3,389, 16 ~ 74 years (Norway)	GAS-7	Birthplace (Norway, Nordic region, Europe, Africa, Asia, North America, South America, Central America, Oceania	Place of birth (Africa, Asia, South- and Middle America) were positively associated with IGD.	Cross-sectional
<School>					
Brunborg et al. (109)	N = 1,928, 13 ~ 17 years (Norway)	GAS-7	Grade Average in Written Norwegian, Mathematics, and English	IGD was associated with poor academic achievement.	Cross-sectional
Wang et al. (110)	N = 503, 14.54 ± 1.42 years for girls; 14.62 ± 1.35 years for boys (Hong Kong)	GAS-7	Social and Demographic Information Self-rated academic performance	IGD was associated with having more than 7 close friends and poor academic performance.	Cross-sectional
* Haghbin et al. (35)	N = 326, high school students (Iran)	GAS-7	Grade Point Average	IGD was associated with poor academic achievement.	Cross-sectional
Richard et al. (111)	N = 6,353, 14.74 ± 1.76 years (USA)	IGDS-SF9	Bullying Victimization PSS	Internalizing and externalizing mediated the relationship between bullying and IGD.	Cross-sectional
<Social support>					
Wartberg et al. (59)	N = 1,095, 12.99 ± 0.82 years (Germany)	Lemmens IGD-9	OSSS	Perceived social support did not predict IGD. High proportion of friends only known through the Internet predicted IGD.	Cross-sectional
Scharkow et al. (112)	N = 4,500, 37.7 years (Germany)	GAS-7	Social capital (i.e., the number of trusted people) Perceived social support	Perceived social support did not predict IGD. Social capital did not predict IGD.	Cross-sectional
* Stockdale and Coyne (50)	Video game addicts: N = 87, 20.80 ± 2.18 years. HC: N = 87 (USA)	Lemmens IGD-9	PROMIS Social Isolation Short Form	Those with IGD felt significantly more socially isolated. There were no differences in feelings of companionship and emotional support.	Cross-sectional
* Uçur and Dönmez (66)	With PIG: N = 144, Without PIG: N = 923, 14.80 ± 1.60 years (Turkey)	GAS-7	MSPSS	IGD was associated with low perceived social support.	Cross-sectional

* Also included in another (psychological) category. ACE = Adverse Childhood Experiences Questionnaire. BSSS = Berlin Social Support Scales. CES-10 = 10 item Center for Epidemiological Studies Short Depression Scale. CSES = Core Self-Evaluations Scale. DASS-21 = Depression ANxiety Stress Scale-21. FoMO = fear of missing out. FRI = Family Relationship Index. GAD-2 = Generalized Anxiety Disorder Scale-2. HC = healthy controls. HSWS = Hikkikomori Social Withdrawal Scale. ICS = Individualism and Collectivism Scale. IPPA = Inventory of Parent and Peer Attachment. ISI = Insomnia Severity Index. MSPSS = Multidimensional scale of perceived social support. OSSS = Oslo Social Support ScalePARQ = Adult Parental Acceptance-Rejection Questionnaire. PHQ-2 = Patient Health Questionnaire-2. PIG = problematic internet gaming. PROMIS = Patient-Reported Outcomes Measurement Information System. PSS = Ohio Scales Youth Problem Severity Scale.

### 3.2. Biological factors associated with IGD

A total of eight studies have investigated biological factors related to GD (Table 1). Among them, three studies investigated reward-related activities and two studies investigated self-concept. The remaining three were specifically related to neurobiology, with one focusing on the brain structure and the other two focusing on functional connectivity (FC).

#### 3.2.1. Reward activity

Using electroencephalography (EEG), one study reported reduced peak amplitudes and longer latencies in response to rewards in pathological computer game players compared to casual players, suggesting a reduced reward sensitivity to gaming rewards in GD (23). Another study using functional magnetic resonance imaging (fMRI) found deficits in the reward and self-control brain systems in response to video gaming cues (24). Specifically, intensive gamers showed stronger activation in the ventral striatum and weaker activation in the dorsolateral prefrontal cortex (DLPFC) compared to controls when watching game-related videos, and GD scores of the gamers were positively associated with the right ventral striatum activity and negatively associated with the right DLPFC activity. Moreover, in a game-deprived state, gamers showed activation in the left insula when exposed to video gaming cues, and the insular activation in the deprivation condition was associated with increased striatal activity and decreased prefrontal activity, which showed similarity to other addictive behaviors. Lower dopamine transporter level has also been associated with more severe GD symptoms in those with GD (22). These studies suggest distinctive functioning of brain regions related to reward in GD.

#### 3.2.2. Self-concept

Two studies consistently reported anomalies in various aspects of self-concept in GD individuals (25, 26). Individuals with GD considered their game avatar significantly superior to their self in terms of social and emotional competencies and they showed hyperactivation in the left angular gyrus (AG), a region that has previously been found to be associated with self-concept-related processing (113, 114), during avatar reflection relative to self-reflection compared to healthy controls. Furthermore, a significant positive correlation was found between the left AG activation and GD severity (26).

#### 3.2.3. Brain structure

GD individuals exhibited decreased gray matter density in the left DLPFC compared to healthy controls and non-problematic game players (27). Moreover, lower gray matter density in the DLPFC was associated with longer lifetime usage of gaming and more severe GD symptoms.

#### 3.2.4. Resting-state fMRI functional connectivity

In an fMRI study that assessed functional connectivity (FC) during resting state, the GD individuals displayed lower FC from the bilateral orbitofrontal cortex to the overall brain (frontal, striatal, temporal, and occipital) regions compared to the healthy controls (29). Another study with a larger sample size reported weaker FC in the central executive network, salience network, and default mode network during resting state in GD individuals compared to healthy controls (28). Specifically, GD individuals showed lower FC between the ventrolateral prefrontal cortex and DLPFC and between the posterior parietal cortex and DLPFC in the central executive network, between the dorsal anterior cingulate cortex and fronto-insular cortex and ventral striatum in the salience network, and in the medial prefrontal cortex of the anterior default mode network compared to healthy controls. Taken together, altered functional connectivity found in these studies might suggest impairments in the capacity of the core brain networks in GD, although more studies are needed to confirm these patterns.

### 3.3. Psychological factors associated with GD

A total of 67 studies have investigated psychological factors related to GD (Table 2). Among them, 24 studies investigated psychiatric symptoms. Seven papers investigated psychological health, with a focus on general well-being. Six investigated emotion regulation and the other five investigated rewards, discounting and impulsivity. Five investigated personality traits, and three investigated stress. Cognitive approach, self-concept, and sleep each included two studies. Considering the current COVID-19 pandemic, a section was designated for COVID-19 which included 8 studies. The remaining studies were categorized as miscellaneous, with one study investigating flow and the other psychological needs.

#### 3.3.1. Psychiatric symptoms

Stockdale and Coyne (50) reported that individuals with GD presented a higher degree of ADHD, anxiety, depression, aggression, and pornography addiction than those without GD. In terms of ADHD, in addition to a bidirectional relationship between GD and ADHD (48), a unidirectional relationship from ADHD to GD (34, 52) as well as from GD to ADHD were found (30, 31) in regression analyses.

In terms of depression and anxiety, a positive association with GD was consistently reported (32, 33, 40–42, 47) with some studies reporting that GD positively predicted the levels of depression and anxiety (36, 54). When focusing on depression or anxiety independently, Severo et al. (45) found a positive association between GD symptoms and depressive symptoms, Singh et al. (46) found that depressive symptoms predicted GD, and Stavropoulos et al. (49) found that GD predicted higher depressive symptoms. One study revealed that depressive symptoms fully mediated the association between children’s emotional trauma and GD (39). In one study, GD scores had positive correlations with anxiety (53) while no association was found in another study (38). Overall, these suggest that GD is associated with ADHD, depression, and anxiety. However, there is a lack of temporal findings to confirm the directionalities.

With regards to other psychiatric symptoms, Musetti et al. (44) found that, compared to non-problematic gamers, problematic gamers displayed higher levels of psychotic symptoms. Murray et al. (43) found that individuals with autism spectrum disorder showed significantly higher GD scores compared to HC. There have been mixed results regarding the substance use patterns, with one study reporting an association between GD severity and polysubstance use (37), while another study reported significantly lower GD symptom scores in individuals with substance use disorder compared to those without the disorder (51).

#### 3.3.2. Psychological health

In one study, GD predicted low self-esteem, perceived social support, and life satisfaction 6 months and 1-year later, suggesting that GD can possibly lead to decreased psychosocial well-being (61). Several studies have found a relationship between GD and antisocial behavior, anger control problems, and hyperactivity and inattention (40, 58, 59, 99). In a Spanish sample, self-esteem, life satisfaction, prosocial behavior, loneliness, and physical aggression predicted GD (55). Among Turkish adults, GD symptoms predicted the presence of lifetime non-suicidal self-injurious behaviors (30, 31). Some studies also found gender effects. Phan et al. (60) found that GD led to decreased quality of life in males, but not in females. Furthermore, the influence of GD tendency on suicide risk was higher in male college students, although female college students showed higher suicide risk on average (56). These results suggest that GD is generally associated with poor psychological health while more studies are needed to confirm gender effects.

#### 3.3.3. Emotion regulation

The inability to manage one’s emotional experience has often been found to be associated with GD (64, 66). Kim and Kwon (63) confirmed that negative emotional experience along with the tendency to play games for mood modification positively predicted GD. Alexithymia, a difficulty describing feelings, and physical aggression also predicted GD symptoms (30, 31). Furthermore, T’ng et al. (65) reported that GD symptoms significantly predicted physical aggression, verbal aggression, anger, and hostility, while Wartberg et al. (62) found that GD significantly predicted subsequent emotional distress 1 year later. These suggest that GD is associated with a poor ability to manage negative emotional responses.

#### 3.3.4. Personality traits

Personality traits are relatively stable characteristics of an individual. The Big Five personality traits are widely used–openness to new experiences, conscientiousness, extraversion, agreeableness, and neuroticism (115). High neuroticism has consistently been found to predict GD while there have been contrasting findings regarding other traits (73, 75, 76). With regards to other personality dimensions, Borzikowsky and Bernhardt (72) found that grit, the perseverance of effort, significantly reduced the GD likelihood, suggesting grit as a potential protective trait against GD. In addition, Müller et al. (74) confirmed that higher scores on maladaptive traits, such as negative affectivity, were significantly associated with GD. These suggest that high neuroticism is associated with GD while more studies are needed to confirm other predisposing traits related to GD.

#### 3.3.5. Reward, discounting, and impulsivity

Wölfling et al. (69) performed a delay discounting task to investigate decision-making in individuals with GD and gambling disorder. They found that the former group showed a faster ability to adapt decision strategies than the latter while there was no significant correlation between GD severity and choice impulsivity. On the other hand, one study found a positive correlation between self-reported impulsivity levels and GD (68). Macur and Pontes (70) reported that gamers with a high GD risk presented significantly lower levels of self-control compared to low-risk gamers or non-gamers. In terms of reward-related decisions, Moudiab and Spada (71) found that overvaluing of gaming rewards predicted GD severity [c.f. (67)]. When combined with biological findings, these suggest that GD is associated with aberrant reward activities and cognition, while a specific pattern is yet to be confirmed.

#### 3.3.6. Stress

All included studies confirmed a positive association between stress and GD (77, 79) and perceived stress predicted more GD symptoms (78).

#### 3.3.7. Cognitive impairment

Bodi et al. (80) found that cognitive salience (e.g., planning what to do next in games) and completion (e.g., feeling the need to achieve objectives as soon as possible) were strong predictors of both online and offline gaming addiction. In addition, Efrati et al. (81) found a positive association between GD and metacognition, an awareness of one’s own thinking, which was mediated by thought suppression. This could signify that a lack of cognitive confidence and beliefs about the need to control thoughts are two metacognitions closely aligned with GD.

#### 3.3.8. Self-concept

Concetta De Pasquale et al. (82) found a positive association between GD and dissociative experiences (e.g., depersonalization and derealization, absorption and imaginative involvement), suggesting that gamers’ predominant immersion in the virtual world could potentially lead to GD. Furthermore, Stavoropoulos et al. (116) found that problematic gaming was more prevalent in gamers highly fused with their game avatars compared to those who successfully differentiated themselves from their avatars. These add to the biological findings that suggest self-concept deficits in GD.

#### 3.3.9. Sleep

Nakayama et al. (84) reported that problematic gamers had significantly later bedtime and wake-up time. Furthermore, Wang et al. (85) found that problematic gaming led individuals to feel that they have poorer sleep quality in general. These suggest the association between poor sleep quality and GD.

#### 3.3.10. Miscellaneous factors

One study has investigated the relationship between GD and psychological flow, defined by the authors as “the feeling of enjoyment and pleasure arising from deep immersion in an activity” (86). They found that flow fully mediated the relationship between the preference for social games and GD, suggesting that GD behaviors may derive from a need to experience flow, especially in a social setting.

Another study has investigated the relationship between GD and need frustration (e.g., when somebody is excluded or rejected by others) (87). They found that game expectancies and time spent on games had a mediating effect on the relationship between the frustration of psychological needs and GD, suggesting that gamers’ need frustration may lead to a greater probability of experiencing GD.

#### 3.3.11. COVID-19

School closures, lockdowns, and social distancing due to the COVID-19 pandemic have profoundly impacted the daily lives of people, leading to increased indoor activities. Time spent on online gaming (91, 93) as well as probable GD prevalence and GD symptoms (90) have constantly increased during the quarantine periods. A longitudinal study found that the levels of video game use and GD severity significantly increased in young populations during the pandemic, and depressive and anxiety symptom scores were associated with such videogame use (91, 92). In addition, COVID-19 related fear, COVID-19 infection status, and psychological distress were found to be associated with GD (90, 93). Loneliness, potentially stemming from the lockdown, was associated with GD behaviors as well (94). Regarding mediating roles, one study reported that GD was a mediator in the association between psychological distress and increased game time during the school hiatus (117). Another study found that psychological distress mediated the relationship between GD and insomnia and quality of life during the pandemic (89). Interestingly, GD behaviors were associated with higher levels of psychological distress only in children who were not overweight (88). These suggest a certain degree of impact COVID-19 has on GD, yet more studies are needed to confirm its long-term effects.

### 3.4. Social factors associated with GD

A total of 29 studies have investigated social factors related to GD (Table 3). Among them, nine studies investigated family-related variables. Eight papers investigated social interactions. Four investigated cultural factors and the other four investigated school-related factors. Lastly, four studies investigated social support.

#### 3.4.1. Family

Bonnaire and Phan (95) found a significantly lower family cohesion, more family conflicts, and a poorer family relationship in problematic gamers compared to non-problematic gamers. Irmak and Erdogen (96) also found that negative family environments predicted GD behaviors but in females only. Sung et al. (101) reported that young adults with adverse childhood experiences had significantly higher GD tendencies compared to those without such experiences. These suggest the link between GD and a family environment and adverse childhood events.

The association between GD and the mental health of family members were often studied together (40, 59, 99) found that self-reported parental anxiety and depression were positively correlated with adolescents’ GD behaviors. Stockdale and Coyne (100) found a direct relationship between problematic gaming and parental efficacy, which was mediated by their depressive symptoms. Lin et al. (98) focused on the mental health of siblings and found that adolescents’ and their siblings’ GD behaviors had significant effects on each other’s depression and anxiety levels. These suggest a role that family members’ mental health plays in the development and maintenance of GD.

Several studies examined the relationship between parenting and GD. In a two-wave study, Koning et al. (97) found that, regardless of gender, GD symptoms predicted more internet-related reactive rules and lower communication quality. Among boys, more frequent internet-related communication predicted GD symptoms. Throuvala et al. (102) found a significant effect of perceived parental rejection on GD symptoms via the mediating factor of low core self-evaluations (e.g., low self-esteem). In a longitudinal study, Teng et al. (103) found that GD negatively predicted children’s subsequent attachment with parents, and the link was stronger in males. These findings suggest a role of parenting on GD, while gender effects are not consistent.

#### 3.4.2. Social interaction

Two studies have reported a significant association between GD and lower levels of sociability as well as less perceived social support (41, 105). Interestingly, Duman and Ozkara (104) found that fear of missing out was a critical predictor of GD. Both the lack of tendency to engage in interpersonal relationships and the need to belong seem to be important factors of GD, although their potential interactions are yet to be studied.

Several studies have investigated social engagements in individuals with GD. In one study, participants were asked “How often do you neglect social contacts because of computer game playing?” and more frequent neglects were found to predict GD (40). Stavropoulos et al. (107) found a positive association between GD and symptoms of Hikikomori, prolonged self-imposed home isolation in addition to avoidance of social engagements. Similarly, De Pasquale et al. (33) reported a significant relationship between GD symptoms as well as a decrease in social relationships and the presence of difficulties in social adaptation. Tullett-Prado et al. (106) found that a high GD risk profile was associated with higher unemployment and a tendency to live with divorced parents or friends. Meanwhile, T’ng et al. (65) found that greater loneliness predicted GD symptoms. These suggest that GD is related to a lack of social engagement and feelings of loneliness.

#### 3.4.3. Culture/ethnicity

Wittek et al. (76) conducted a national survey in Norway and found that individuals born in Africa, Asia, South- or Central America were 4.9 times more likely to belong to the GD group compared to those born in Norway. *While it could be inferred that the participants from races/cultures mentioned in the study might experience a higher degree of GD as a result of their culture or race, it is equally plausible that these experiences are more attributable to the challenges they face in being accepted within the host culture*. In terms of cultural orientations, Andreetta et al. (77) and Stavropoulos et al. (83) found a positive association between GD symptoms and vertical individualism, suggesting that gamers who endorse more individualistic cultural orientation potentially have a higher risk of GD. Similarly Stavropoulos et al. (49, 108), reported that gamers aversive to collectivism displayed more GD behaviors and addiction-related symptoms (e.g., withdrawal) compared to those who were neutral. These imply that individualistic cultural orientation is more related to GD compared to collectivism, while its association with specific ethnic groups is yet to be found.

#### 3.4.4. School

Haghbin et al. (35) reported a significant negative relationship between GD and high school students’ grade point average. Along the same lines, in a two-wave longitudinal study, Brunborg et al. (109) found that GD at time point 1 was negatively correlated with academic achievement both at time points 1 and 2. Specifically, a 10% increase in GD symptoms was associated with a 1.7 point decrease in average grades. Wang et al. (110) also found that children with poor self-reported academic performance were significantly more likely to have GD compared to those with good academic performance.

In addition to academics, peer relations take up a significant proportion of children and adolescents’ school life. In the above study, they found that children who reported having more friends (7 or more) were more likely to have GD than others. On the other hand, Richard et al. (111) reported a significant relationship between bullying experiences and GD. These suggest that poor academic achievements are consistently found in GD, but the effect of peer relations needs further investigation.

#### 3.4.5. Social support

No significant interdependencies between GD and perceived social support nor social capital was found (59, 112). Similarly, Stockdale and Coyne (50) found no differences in social support or feelings of companionship between individuals with and without GD. Contrary to these null findings, Ucur and Donmez (66) found that perceived social support was significantly lower in adolescents with GD. These suggest that there is little evidence that GD is related to negative changes in psychosocial status.

## 4. Discussion and conclusion

Using the PRISMA guidelines (19), we performed a systematic review to provide an up-to-date, comprehensive review of the empirical evidence of GD. We explored biopsychosocial factors of GD by systematically identifying studies that have utilized one of five GD screening tools that possess greater evidential support. Our review elucidated various factors in the biological, psychological, and social domains that were associated with GD. On the other hand, we have identified critical gaps in the literature related to study designs and assessment tool usage, in a way that most were cross-sectional and have utilized GD assessment tools with varying properties.

Notwithstanding the importance of neurobiological explorations in understanding a psychiatric condition, biological mechanisms underlying GD are relatively poorly understood (118, 119). Studies in the review compared reward activation, self-concept, brain structure, and functional connectivity between individuals with and without GD. GD was generally associated with reduced reward sensitivity to gaming-related rewards, heightened activation in the reward-related brain regions to gaming-related cues, and deficiencies in dopaminergic activities. However, one study included in the review suggested the absence of an association between reward sensitivity and gaming behaviors but rather found its association with harmful smartphone use, suggesting a possible effect of gaming method when investigating reward-related behaviors (67). These different results were in line with previous studies that have reported conflicting results regarding reward-related activations in individuals with GD (120, 121). In addition, reward networks in individuals with GD and those with other addictive disorders also yielded inconsistent results (122, 123). Although many studies in the review suggest certain reward patterns in GD, the lack of studies makes it difficult to draw a conclusion regarding the resemblance of GD to other well-defined addictive disorders.

In terms of self-concept in GD, the gamers’ tendency to identify with their game avatars increased as they transitioned from normal to problematic users. This follows the past review that reported self-concept deficits and increased identification with the gaming character in individuals with GD (124), further highlighting the discrepancy between the real self and the virtual self in GD. With regards to brain structure and connectivity, lower gray matter density in the left DLPFC and lower FC between bilateral orbitofrontal cortex and overall brain regions were observed but due to the lack of studies included, it is premature to establish these as a general pattern of the GD.

Most of the studies have investigated psychological aspects of GD, suggesting abnormalities in multiple domains. The most robust association was found between GD and other psychiatric conditions (e.g., ADHD, depression, and anxiety). Studies investigating the effect of COVID-19 on GD have also discovered a significant role depression and anxiety play in GD. This is in accordance with previous studies that have proposed comorbidities between GD and a range of psychological symptoms including anxiety, depression, and attention problems, both in adults and adolescents (125–127). While this could be suggestive of the relevant impact that mood disturbances and attention problems have on GD, this also raises a question as to whether GD possesses a unique profile or whether the symptoms stem from underlying conditions (128, 129). While a mere existence of comorbidities does not automatically explain the observed health conditions better, this still highlights the need to control for comorbidities when investigating unique features of the GD. In terms of overall psychological health, GD was associated with low self-esteem, low life satisfaction, high suicidality, high maladaptive personality traits, and high levels of stress yet direct casualties could not be drawn due to the cross-sectional designs of the studies.

Differential results were found with regards to cognitive approach and patterns. While higher impulsivity and maladaptive cognition were found to be associated with GD in one study (68), another study had found no such association and even found that individuals with GD adopted more advantageous decision strategies compared to those with gambling disorder or healthy individuals (69). The lack of consistency in the results contradicts many GD models that emphasize higher impulsivity and difficulties of decision-making (17, 130) as important features of GD. This warrants more studies to settle the accuracy of these models, especially since impulsivity and abnormal decision-making processes are considered significant features of addiction (131, 132).

For social factors, family members’ mental health, individualistic cultural orientation, and poor academic achievement were consistently found to be associated with GD. On the other hand, some noticeable variabilities were observed, specifically with regards to family-related factors. This included certain gender effects in terms of how family rules and attachment affect children’s GD symptoms as well as the directionality of the association between GD and family issues. Furthermore, it could not be concluded whether the family issues directly affect children’s GD, or whether there is a mediating factor concerning the traits of the children themselves. The contributions of social support and extra-family relationships to GD symptoms also yielded mixed results (e.g., having many friends vs. neglect of social contacts, low social support vs. no significant differences), making it difficult to conceptualize the role of social factors in the development and maintenance of GD. As family factors hugely impact children’s and adolescents’ development, studies should track temporal patterns of GD symptoms in order to unravel the complicated relationships (105).

Several gaps in the field of GD research were detected in the present review. First, more longitudinal and long-term follow-up studies are needed. Most studies included in this review were cross-sectional thus causal relationships nor predictive functions of each biopsychosocial variable could not be drawn. As mere correlations are not adequate to support a formalization of a disorder, a more sophisticated and deeper level of evidence of GD is needed (133). As one way to examine longitudinal aspects of gaming, we advise researchers to actively utilize large-scale projects, such as the ABCD [Adolescent Brain and Cognitive Development; (134)] study or the Project M.E.D.I.A (Media Effects on Development from Infancy to Adulthood; https://www.projectmediadenver.com/) to name a few, which are ongoing, longitudinal studies on child development.

Second, standardized approaches to GD assessment tools are essential. The problems that arise from the variability in GD screening tools in addition to the need for a unified approach have been highlighted by many researchers (13, 14, 135, 136). Even though we have made an attempt to control for these issues by only including studies that have utilized one of five scales with greater evidential support, we could not fully rule out the differences that exist between these five scales. Therefore, rather than constantly developing new diagnostic tools with varying conceptual properties, assessment consistency should initially be established to improve the comparability of different research studies.

Third, it was apparent from the review that there were not enough studies that have investigated biological factors of GD using screening tools of choice. During the literature search, we found that scales developed to measure general internet addiction rather than GD were being widely used in studies that collected neural and behavioral data, while it has constantly been argued that internet addiction should be conceptually and clinically distinguished from GD (137, 138). More studies focusing on the neurobiology and genetics of GD while using screening tools with evidential support or clinical interview are needed to uncover its underlying biological mechanisms. This will allow researchers to gain insight into the biological mechanisms, especially since animal models that have facilitated understanding of substance use disorders are lacking for GD (139).

Lastly, game-related factors (e.g., game genres, game time) should be taken into consideration when designing studies. It has been argued that the ‘social’ aspect of gaming is what leads to various problems instead of ‘gaming’ itself (140). As mixed and varying results were observed with regards to social factors, future studies should control for game genres as much as possible. Furthermore, there have been contrasting results regarding whether the amount of game time could be considered a reliable predictor of GD, questioning the idea that intense gaming itself is problematic (141). Colder Carras and Kardefelt-Winther (142) have also raised concerns that highly involved gamers could be misclassified simply due to the time they engage in gaming. Future studies should examine the association between game time and GD for more accurate clinical diagnostic criteria.

In conclusion, this was the first systematic review on GD to control for the excess amount of assessment tools with varying psychometric properties to provide an up-to-date and comprehensive overview of biopsychosocial factors associated with GD. While several biological, psychological, and social factors--impaired self-concept, comorbidities, emotional dysregulation, and poor academic performance--were consistently confirmed, mixed results were observed mainly with regards to reward activities, impulsivity, social relations, and family relationships. To settle the debate in terms of psychometric weaknesses of the current diagnostic system, collaborative approaches among experts in education, mental health, and the gaming industry seem crucial (143, 144). We conclude that more longitudinal and neurobiological studies, consensus on a diagnostic tool with well-defined psychometric properties, and an in-depth understanding of gaming-related factors should be established.

## Author contributions

RC: conceptualization, methodology, investigation, resources, data curation, writing – original draft, writing – review and editing, visualization, and project administration. ML: conceptualization, methodology, investigation, resources, data curation, writing – review and editing, and visualization. JI: investigation, resources, data curation, writing – review and editing, and visualization. K-HC, JK, JC, and S-HS: writing – review and editing. W-YA: conceptualization, methodology, writing – review and editing, and funding acquisition. All authors contributed to the article and approved the submitted version.

## Funding

This work was supported by the Ministry of Health and Welfare, Republic of Korea and the Ministry of Culture, Sports and Tourism, Republic of Korea, a grant from the National Research Foundation (NRF) of Korea funded by the Korean government (NRF-2018R1C1B3007313), and the Creative-Pioneering Researchers Program through Seoul National University (W-YA).

## Conflict of interest

The authors declare that the research was conducted in the absence of any commercial or financial relationships that could be construed as a potential conflict of interest.

## Publisher’s note

All claims expressed in this article are solely those of the authors and do not necessarily represent those of their affiliated organizations, or those of the publisher, the editors and the reviewers. Any product that may be evaluated in this article, or claim that may be made by its manufacturer, is not guaranteed or endorsed by the publisher.
