# Supplementary material for: Biopsychosocial factors of gaming disorder: a systematic review employing screening tools with well-defined psychometric properties
Source: Front Psychiatry. 2023 Jul 18;14:1200230. doi: 10.3389/fpsyt.2023.1200230 (PMC10390702; doi:10.3389/fpsyt.2023.1200230)
Supplement: Supplementary file 1 [file Data_Sheet_1.docx]

**Supplementary material:**

- **Appendix A.** Kmet quantitative analysis for included studies
- **Appendix B.** Descriptions for five diagnostic tools

**Appendix A. Kmet quantitative analysis for included studies**

(a) Kmet analysis for studies included in the biological category (YES (2), PARTIAL (1), NO (0), N/A)

| Criteria | QUESTION | Ariatama, Effendy, & Amin (2019) | Choi et al. (2017) | Chun et al. (2020) | Dieter et al. (2015) | Duven, Müller, Beutel, & Wölfling (2015) | Kim et al. (2019) | Lemenager et al. (2016) | Turel, He, Wei, & Bechara (2021) |  |
| --- | --- | --- | --- | --- | --- | --- | --- | --- | --- | --- |
| 1 | Question/objective sufficiently described? | 2 | 2 | 2 | 2 | 2 | 2 | 2 | 2 |  |
| 2 | Study design evident and appropriate? | 2 | 2 | 2 | 2 | 2 | 2 | 2 | 2 |  |
| 3 | Method of subject/comparison group selection or source of information/input variables described and appropriate? | 2 | 2 | 2 | 2 | 2 | 2 | 2 | 2 |  |
| 4 | Subject (and comparison group, if applicable) characteristics sufficiently described? | 2 | 2 | 2 | 2 | 2 | 2 | 2 | 2 |  |
| 5 | If interventional and random allocation was possible, was it described? | N/A | N/A | N/A | N/A | N/A | N/A | N/A | N/A |  |
| 6 | If interventional and blinding of investigators was possible, was it reported? | N/A | N/A | N/A | N/A | N/A | N/A | N/A | N/A |  |
| 7 | If interventional and blinding of subjects was possible, was it reported? | N/A | N/A | N/A | N/A | N/A | N/A | N/A | N/A |  |
| 8 | Outcome and (if applicable) exposure measure(s) well defined and robust to measurement/misclassification bias? Means of assessment reported? | 2 | 2 | 2 | 2 | 2 | 2 | 2 | 2 |  |
| 9 | Sample size appropriate? | 2 | 2 | 2 | 1 | 1 | 2 | 1 | 2 |  |
| 10 | Analytic methods described/justified and appropriate? | 2 | 2 | 2 | 2 | 2 | 2 | 2 | 2 |  |
| 11 | Some estimate of variance is reported for the main results? | 0 | 2 | 2 | 2 | 2 | 2 | 2 | 2 |  |
| 12 | Controlled for confounding? | 0 | 2 | 2 | 2 | 1 | 2 | 1 | 2 |  |
| 13 | Results reported in sufficient detail? | 1 | 2 | 2 | 2 | 2 | 2 | 2 | 2 |  |
| 14 | Conclusions supported by the results? | 2 | 2 | 2 | 2 | 2 | 2 | 2 | 2 |  |
|  | **Total sum** | **17** | **22** | **22** | **21** | **20** | **21** | **20** | **22** |  |
|  | **Total possible sum** | **22** | **22** | **22** | **22** | **22** | **22** | **22** | **22** |  |
|  | **Summary Score (total sum / total possible sum)** | **0.77** | **1.00** | **1.00** | **0.95** | **0.91** | **0.95** | **0.91** | **1.00** |  |

(b) Kmet analysis for studies included in both psychological and social categories (YES (2), PARTIAL (1), NO (0), N/A)

| Criteria | QUESTION | Andreetta, Teh, Burleigh, Gomez, & Stavropoulos (2020) | De Pasquale et al. (2020) | Haghbin, Shaterian, Hosseinzadeh, & Griffiths (2013) | Männikkö, Billieux, & Kääriäinen (2015) | Stockdale & Coyne (2018) | | T’ng, Ho, Sim, Yu, & Wong (2020) | Uçur & Dönmez (2021) | Wartberget al. (2017) | Wartberg, Kriston, & Thomasius (2017) | Wittek et al. (2016) |
| --- | --- | --- | --- | --- | --- | --- | --- | --- | --- | --- | --- | --- |
| 1 | Question/objective sufficiently described? | 2 | 1 | 2 | 2 | 2 | 2 | | 2 | 1 | 1 | 2 |
| 2 | Study design evident and appropriate? | 2 | 2 | 2 | 2 | 2 | 2 | | 2 | 2 | 2 | 2 |
| 3 | Method of subject/comparison group selection or source of information/input variables described and appropriate? | 1 | 2 | 2 | 2 | 2 | 2 | | 1 | 1 | 2 | 2 |
| 4 | Subject (and comparison group, if applicable) characteristics sufficiently described? | 2 | 2 | 1 | 2 | 2 | 2 | | 2 | 2 | 2 | 2 |
| 5 | If interventional and random allocation was possible, was it described? | N/A | N/A | N/A | N/A | N/A | N/A | | N/A | N/A | N/A | N/A |
| 6 | If interventional and blinding of investigators was possible, was it reported? | N/A | N/A | N/A | N/A | N/A | N/A | | N/A | N/A | N/A | N/A |
| 7 | If interventional and blinding of subjects was possible, was it reported? | N/A | N/A | N/A | N/A | N/A | N/A | | N/A | N/A | N/A | N/A |
| 8 | Outcome and (if applicable) exposure measure(s) well defined and robust to measurement/misclassification bias? Means of assessment reported? | 2 | 2 | 2 | 2 | 2 | 2 | | 2 | 2 | 2 | 2 |
| 9 | Sample size appropriate? | N/A | N/A | N/A | 1 | 2 | N/A | | 1 | N/A | N/A | 1 |
| 10 | Analytic methods described/justified and appropriate? | 2 | 2 | 2 | 2 | 2 | 2 | | 2 | 2 | 2 | 2 |
| 11 | Some estimate of variance is reported for the main results? | 2 | 0 | 2 | 2 | 2 | 2 | | 2 | 2 | 2 | 2 |
| 12 | Controlled for confounding? | 2 | 0 | 0 | 2 | 2 | 0 | | 2 | 2 | 2 | 2 |
| 13 | Results reported in sufficient detail? | 2 | 2 | 2 | 2 | 2 | 2 | | 2 | 2 | 2 | 2 |
| 14 | Conclusions supported by the results? | 2 | 2 | 1 | 2 | 1 | 2 | | 2 | 2 | 2 | 2 |
|  | **Total sum** | **19** | **15** | **16** | **21** | **21** | **18** | | **20** | **18** | **19** | **21** |
|  | **Total possible sum** | **20** | **20** | **20** | **22** | **22** | **20** | | **22** | **20** | **20** | **22** |
|  | **Summary Score (total sum / total possible sum)** | **0.95** | **0.75** | **0.80** | **0.95** | **0.95** | **0.90** | | **0.91** | **0.90** | **0.95** | **0.95** |

(c) Kmet analysis for studies included in the psychological category (YES (2), PARTIAL (1), NO (0), N/A)

| Criteria | QUESTION | Acuff et al. (2021) | Bodi, Maintenant, & Pennequin (2021) | Bonnaire & Baptista (2019) | Borzikowsky & Bernhardt (2018) | Buiza-Aguado, Alonso-Canovas, Conde-Mateos, Buiza-Navarrete, & Gentile (2018) | Canale et al. (2019) | Cerniglia et al. (2019) | Chamarro, Oberst, Cladellas, & Fuster (2020) | Chen, Chen, O’Brien, Latner, & Lin (2021) | Chen, Chen, Pakpour, Lin, & Griffiths (2021) | Choi (2018) |
| --- | --- | --- | --- | --- | --- | --- | --- | --- | --- | --- | --- | --- |
| 1 | Question/objective sufficiently described? | 2 | 2 | 2 | 2 | 2 | 2 | 2 | 2 | 2 | 2 | 2 |
| 2 | Study design evident and appropriate? | 2 | 2 | 2 | 2 | 2 | 2 | 2 | 2 | 2 | 2 | 2 |
| 3 | Method of subject/comparison group selection or source of information/input variables described and appropriate? | 2 | 2 | 2 | 2 | 2 | 2 | 2 | 2 | 2 | 2 | 2 |
| 4 | Subject (and comparison group, if applicable) characteristics sufficiently described? | 2 | 2 | 2 | 2 | 2 | 1 | 2 | 2 | 1 | 2 | 2 |
| 5 | If interventional and random allocation was possible, was it described? | N/A | N/A | N/A | N/A | N/A | N/A | N/A | N/A | N/A | N/A | N/A |
| 6 | If interventional and blinding of investigators was possible, was it reported? | N/A | N/A | N/A | N/A | N/A | N/A | N/A | N/A | N/A | N/A | N/A |
| 7 | If interventional and blinding of subjects was possible, was it reported? | N/A | N/A | N/A | N/A | N/A | N/A | N/A | N/A | N/A | N/A | N/A |
| 8 | Outcome and (if applicable) exposure measure(s) well defined and robust to measurement/misclassification bias? Means of assessment reported? | 2 | 2 | 2 | 2 | 2 | 2 | 2 | 2 | 2 | 2 | 2 |
| 9 | Sample size appropriate? | N/A | 1 | 1 | N/A | 1 | N/A | N/A | N/A | 2 | 2 | 2 |
| 10 | Analytic methods described/justified and appropriate? | 2 | 2 | 2 | 2 | 2 | 2 | 2 | 2 | 2 | 2 | 2 |
| 11 | Some estimate of variance is reported for the main results? | 2 | 2 | 2 | 2 | 2 | 0 | 0 | 2 | 2 | 2 | 2 |
| 12 | Controlled for confounding? | 2 | 1 | 2 | 2 | 1 | 2 | 2 | 2 | 1 | 1 | 1 |
| 13 | Results reported in sufficient detail? | 2 | 1 | 2 | 2 | 1 | 2 | 2 | 2 | 2 | 2 | 2 |
| 14 | Conclusions supported by the results? | 2 | 2 | 2 | 2 | 2 | 2 | 2 | 2 | 2 | 2 | 2 |
|  | **Total sum** | **18** | **19** | **21** | **20** | **19** | **17** | **18** | **20** | **20** | **21** | **21** |
|  | **Total possible sum** | **20** | **22** | **22** | **20** | **22** | **20** | **20** | **20** | **22** | **22** | **22** |
|  | **Summary Score (total sum / total possible sum)** | **0.90** | **0.86** | **0.95** | **1.00** | **0.86** | **0.85** | **0.90** | **1.00** | **0.91** | **0.95** | **0.95** |

| Criteria | QUESTION | Concerto et al. (2021) | De Pasquale, Dinaro, & Sciacca (2018) | Efrati, Kolubinski, Marino, & Spada (2021) | Evren, Evren, Dalbudak, Topcu, & Kutlu (2019) | Evren et al. (2019) | Evren et al. (2020) | Fazeli et al. (2020) | Horváth et al. (2021) | Hu, Stavropoulos, Anderson, Scerri, & Collard (2019) | Ismail et al. (2021) | Kim, Jung, & Cho (2021) | Kim & Kwon (2018) |  |  |
| --- | --- | --- | --- | --- | --- | --- | --- | --- | --- | --- | --- | --- | --- | --- | --- |
| 1 | Question/objective sufficiently described? | 2 | 1 | 2 | 2 | 2 | 2 | 2 | 2 | 2 | 2 | 2 | 2 |  |  |
| 2 | Study design evident and appropriate? | 2 | 2 | 2 | 2 | 2 | 2 | 2 | 2 | 2 | 1 | 2 | 2 |  |  |
| 3 | Method of subject/comparison group selection or source of information/input variables described and appropriate? | 2 | 2 | 1 | 1 | 2 | 2 | 2 | 2 | 2 | 2 | 2 | 2 |  |  |
| 4 | Subject (and comparison group, if applicable) characteristics sufficiently described? | 2 | 1 | 2 | 2 | 2 | 2 | 2 | 2 | 2 | 2 | 1 | 2 |  |  |
| 5 | If interventional and random allocation was possible, was it described? | N/A | N/A | N/A | N/A | N/A | N/A | N/A | N/A | N/A | N/A | N/A | N/A |  |  |
| 6 | If interventional and blinding of investigators was possible, was it reported? | N/A | N/A | N/A | N/A | N/A | N/A | N/A | N/A | N/A | N/A | N/A | N/A |  |  |
| 7 | If interventional and blinding of subjects was possible, was it reported? | N/A | N/A | N/A | N/A | N/A | N/A | N/A | N/A | N/A | N/A | N/A | N/A |  |  |
| 8 | Outcome and (if applicable) exposure measure(s) well defined and robust to measurement/misclassification bias? Means of assessment reported? | 2 | 2 | 2 | 2 | 2 | 2 | 2 | 2 | 2 | 2 | 2 | 2 |  |  |
| 9 | Sample size appropriate? | N/A | N/A | 2 | N/A | 2 | N/A | 2 | 2 | N/A | 2 | 1 | 1 |  |  |
| 10 | Analytic methods described/justified and appropriate? | 2 | 2 | 2 | 2 | 2 | 2 | 2 | 2 | 2 | 1 | 2 | 2 |  |  |
| 11 | Some estimate of variance is reported for the main results? | 2 | 2 | 2 | 1 | 2 | 2 | 2 | 2 | 2 | 2 | 1 | 2 |  |  |
| 12 | Controlled for confounding? | 2 | 1 | 2 | 2 | 2 | 2 | 2 | 2 | 2 | 1 | 1 | 2 |  |  |
| 13 | Results reported in sufficient detail? | 2 | 2 | 1 | 1 | 2 | 1 | 2 | 2 | 2 | 2 | 2 | 2 |  |  |
| 14 | Conclusions supported by the results? | 2 | 2 | 2 | 2 | 2 | 2 | 2 | 2 | 2 | 1 | 2 | 2 |  |  |
|  | **Total sum** | **20** | **17** | **20** | **17** | **22** | **19** | **22** | **22** | **20** | **18** | **18** | **21** |  |  |
|  | **Total possible sum** | **20** | **20** | **22** | **20** | **22** | **20** | **22** | **22** | **20** | **22** | **22** | **22** |  |  |
|  | **Summary Score (total sum / total possible sum)** | **1.00** | **0.85** | **0.91** | **0.85** | **1.00** | **0.95** | **1.00** | **1.00** | **1.00** | **0.82** | **0.82** | **0.95** |  |  |

| Criteria | QUESTION | Kagan Kircaburun, Griffiths, & Billieux (2019) | Macur & Pontes (2021) | Mentzoni et al. (2011) | Moudiab & Spada (2019) | Müller & Bonnaire (2021) | Müller, Beutel, Egloff, & Wölfling (2014) | Müller, Werthmann, Beutel, Wölfling (2021) | Murray, Mannion, Chen, & Leader (2021) | Musetti et al. (2019) | Nakayama, Matsuzaki, Mihara, Kitayuguchi, & Higuchi (2020) | Oka et al. (2021) | Phan, Prieur, Bonnaire, & Obradovic (2020) |  |  |
| --- | --- | --- | --- | --- | --- | --- | --- | --- | --- | --- | --- | --- | --- | --- | --- |
| 1 | Question/objective sufficiently described? | 1 | 2 | 2 | 1 | 2 | 2 | 2 | 2 | 2 | 2 | 2 | 2 |  |  |
| 2 | Study design evident and appropriate? | 2 | 2 | 2 | 2 | 2 | 2 | 2 | 2 | 2 | 2 | 2 | 2 |  |  |
| 3 | Method of subject/comparison group selection or source of information/input variables described and appropriate? | 2 | 2 | 2 | 2 | 2 | 2 | 2 | 2 | 2 | 2 | 2 | 2 |  |  |
| 4 | Subject (and comparison group, if applicable) characteristics sufficiently described? | 2 | 2 | 2 | 2 | 2 | 2 | 2 | 2 | 2 | 2 | 2 | 2 |  |  |
| 5 | If interventional and random allocation was possible, was it described? | N/A | N/A | N/A | N/A | N/A | N/A | N/A | N/A | N/A | N/A | N/A | N/A |  |  |
| 6 | If interventional and blinding of investigators was possible, was it reported? | N/A | N/A | N/A | N/A | N/A | N/A | N/A | N/A | N/A | N/A | N/A | N/A |  |  |
| 7 | If interventional and blinding of subjects was possible, was it reported? | N/A | N/A | N/A | N/A | N/A | N/A | N/A | N/A | N/A | N/A | N/A | N/A |  |  |
| 8 | Outcome and (if applicable) exposure measure(s) well defined and robust to measurement/misclassification bias? Means of assessment reported? | 2 | 2 | 2 | 2 | 2 | 2 | 2 | 2 | 2 | 2 | 2 | 2 |  |  |
| 9 | Sample size appropriate? | N/A | 1 | N/A | N/A | 1 | 2 | 2 | 2 | 1 | 1 | 2 | 2 |  |  |
| 10 | Analytic methods described/justified and appropriate? | 2 | 2 | 2 | 2 | 2 | 2 | 2 | 2 | 2 | 2 | 1 | 2 |  |  |
| 11 | Some estimate of variance is reported for the main results? | 2 | 2 | 2 | 1 | 2 | 2 | 2 | 2 | 2 | 2 | 2 | 2 |  |  |
| 12 | Controlled for confounding? | 2 | 2 | 2 | 2 | 1 | 2 | 2 | 2 | 2 | 1 | 2 | 2 |  |  |
| 13 | Results reported in sufficient detail? | 1 | 2 | 2 | 1 | 2 | 2 | 2 | 2 | 2 | 1 | 2 | 2 |  |  |
| 14 | Conclusions supported by the results? | 2 | 2 | 2 | 2 | 2 | 2 | 2 | 2 | 2 | 2 | 1 | 2 |  |  |
|  | **Total sum** | **18** | **21** | **20** | **17** | **20** | **22** | **22** | **22** | **21** | **19** | **20** | **22** |  |  |
|  | **Total possible sum** | **20** | **22** | **20** | **20** | **22** | **22** | **22** | **22** | **22** | **22** | **22** | **22** |  |  |
|  | **Summary Score (total sum / total possible sum)** | **0.90** | **0.95** | **1.00** | **0.85** | **0.91** | **1.00** | **1.00** | **1.00** | **0.95** | **0.86** | **0.91** | **1.00** |  |  |

| Criteria | QUESTION | Pontes (2017) | Rajab et al. (2020) | Sallie, Ritou, Bowden-Jones, & Voon (2021) | Sánches-llorens et al. (2021) | Severo et al. (2020) | Singh, Dahiya, Singh, Kumar, & Balhara (2019) | Siste et al. (2021) | Stavropoulos et al. (2019) | Stavropoulos, Gomez, Mueller, Yucel, & Griffiths (2020) | Stavropoulos, Vassallo, Burleigh, Gomez, & Colder Carras (2021) | Zhaojun Teng, Pontes, et al. (2020) | Ting & Essau (2021) |  |  |  |  |
| --- | --- | --- | --- | --- | --- | --- | --- | --- | --- | --- | --- | --- | --- | --- | --- | --- | --- |
| 1 | Question/objective sufficiently described? | 2 | 2 | 2 | 2 | 1 | 1 | 1 | 2 | 2 | 2 | 2 | 2 |  |  |  |  |
| 2 | Study design evident and appropriate? | 2 | 2 | 2 | 2 | 2 | 2 | 2 | 2 | 2 | 2 | 2 | 2 |  |  |  |  |
| 3 | Method of subject/comparison group selection or source of information/input variables described and appropriate? | 2 | 2 | 2 | 2 | 2 | 2 | 2 | 1 | 0 | 2 | 2 | 2 |  |  |  |  |
| 4 | Subject (and comparison group, if applicable) characteristics sufficiently described? | 2 | 2 | 2 | 2 | 2 | 2 | 2 | 2 | 2 | 2 | 2 | 2 |  |  |  |  |
| 5 | If interventional and random allocation was possible, was it described? | N/A | N/A | N/A | N/A | N/A | N/A | N/A | N/A | N/A | N/A | N/A | N/A |  |  |  |  |
| 6 | If interventional and blinding of investigators was possible, was it reported? | N/A | N/A | N/A | N/A | N/A | N/A | N/A | N/A | N/A | N/A | N/A | N/A |  |  |  |  |
| 7 | If interventional and blinding of subjects was possible, was it reported? | N/A | N/A | N/A | N/A | N/A | N/A | N/A | N/A | N/A | N/A | N/A | N/A |  |  |  |  |
| 8 | Outcome and (if applicable) exposure measure(s) well defined and robust to measurement/misclassification bias? Means of assessment reported? | 2 | 2 | 2 | 2 | 2 | 2 | 2 | 2 | 2 | 2 | 2 | 2 |  |  |  |  |
| 9 | Sample size appropriate? | N/A | 2 | 2 | 2 | N/A | N/A | N/A | N/A | 2 | N/A | 2 | 2 |  |  |  |  |
| 10 | Analytic methods described/justified and appropriate? | 2 | 2 | 2 | 1 | 2 | 2 | 2 | 2 | 2 | 2 | 2 | 2 |  |  |  |  |
| 11 | Some estimate of variance is reported for the main results? | 2 | 2 | 2 | 2 | 2 | 2 | 2 | 2 | 2 | 2 | 2 | 2 |  |  |  |  |
| 12 | Controlled for confounding? | 1 | 2 | 2 | 1 | 1 | 2 | 2 | 2 | 2 | 2 | 2 | 2 |  |  |  |  |
| 13 | Results reported in sufficient detail? | 2 | 2 | 2 | 2 | 1 | 2 | 2 | 2 | 2 | 2 | 2 | 2 |  |  |  |  |
| 14 | Conclusions supported by the results? | 2 | 2 | 2 | 2 | 1 | 2 | 2 | 2 | 2 | 2 | 2 | 2 |  |  |  |  |
|  | **Total sum** | **19** | **22** | **22** | **20** | **16** | **19** | **19** | **19** | **20** | **20** | **22** | **22** |  |  |  |  |
|  | **Total possible sum** | **20** | **22** | **22** | **22** | **20** | **20** | **20** | **20** | **22** | **20** | **22** | **22** |  |  |  |  |
|  | **Summary Score (total sum / total possible sum)** | **0.95** | **1.00** | **1.00** | **0.91** | **0.80** | **0.95** | **0.95** | **0.95** | **0.91** | **1.00** | **1.00** | **1.00** |  |  |  |  |

| Criteria | QUESTION | Teng, Pontes, Nie, Griffiths, & Guo (2021) | Turhan Gürbüz et al. (2021) | Vally (2021) | Wang, Mati, & Cai (2021) | Wartberg, Kriston, Zieglmeier, Lincoln, & Kammerl (2019) | Wartberg, Zieglmeier, & Kammerl (2021) | Wölfling, Duven, Wejbera, Beutel, & Müller (2020) | Wong et al. (2020) | Zhu, Zhuang, Lee, Li, & Wong (2021) |
| --- | --- | --- | --- | --- | --- | --- | --- | --- | --- | --- |
| 1 | Question/objective sufficiently described? | 2 | 2 | 2 | 2 | 2 | 2 | 2 | 2 | 2 |
| 2 | Study design evident and appropriate? | 2 | 2 | 2 | 2 | 2 | 2 | 2 | 2 | 2 |
| 3 | Method of subject/comparison group selection or source of information/input variables described and appropriate? | 2 | 2 | 2 | 2 | 1 | 1 | 2 | 2 | 2 |
| 4 | Subject (and comparison group, if applicable) characteristics sufficiently described? | 2 | 2 | 2 | 2 | 1 | 2 | 2 | 2 | 2 |
| 5 | If interventional and random allocation was possible, was it described? | N/A | N/A | N/A | N/A | N/A | N/A | N/A | N/A | N/A |
| 6 | If interventional and blinding of investigators was possible, was it reported? | N/A | N/A | N/A | N/A | N/A | N/A | N/A | N/A | N/A |
| 7 | If interventional and blinding of subjects was possible, was it reported? | N/A | N/A | N/A | N/A | N/A | N/A | N/A | N/A | N/A |
| 8 | Outcome and (if applicable) exposure measure(s) well defined and robust to measurement/misclassification bias? Means of assessment reported? | 2 | 2 | 2 | 2 | 2 | 2 | 2 | 2 | 2 |
| 9 | Sample size appropriate? | 2 | 2 | N/A | N/A | 2 | 1 | N/A | 2 | 2 |
| 10 | Analytic methods described/justified and appropriate? | 2 | 2 | 2 | 2 | 2 | 2 | 2 | 2 | 2 |
| 11 | Some estimate of variance is reported for the main results? | 2 | 2 | 2 | 2 | 2 | 2 | 2 | 2 | 2 |
| 12 | Controlled for confounding? | 2 | 1 | 2 | 2 | 2 | 1 | 2 | 2 | 2 |
| 13 | Results reported in sufficient detail? | 2 | 2 | 1 | 2 | 2 | 1 | 2 | 2 | 2 |
| 14 | Conclusions supported by the results? | 2 | 2 | 2 | 2 | 2 | 2 | 2 | 2 | 2 |
|  | **Total sum** | **22** | **21** | **19** | **20** | **20** | **18** | **20** | **22** | **22** |
|  | **Total possible sum** | **22** | **22** | **20** | **20** | **22** | **22** | **20** | **22** | **22** |
|  | **Summary Score (total sum / total possible sum)** | **1.00** | **0.95** | **0.95** | **1.00** | **0.91** | **0.82** | **1.00** | **1.00** | **1.00** |

(d) Kmet analysis for studies included in the social category (YES (2), PARTIAL (1), NO (0), N/A)

| Criteria | QUESTION | Bonnaire & Phan (2017) | Brunborg, Mentzoni, & Frøyland (2014) | Duman & Ozkara (2019) | Festl, Scharkow, & Quandt (2012) | Irmak & Erdogan (2019) | Koning, Peeters, Finkenauer, & Van Den Eijinden (2018) | Lin, Potenza, Broström, & Pakpour (2021) | Richard, Marchica, Ivoska, & Derevensky (2021) | Scharkow, Festl, & Quandt (2014) | Zhaojun Teng, Griffiths, Nie, Ziang, & Guo (2020) | Stavropoulos et al. (2019) |
| --- | --- | --- | --- | --- | --- | --- | --- | --- | --- | --- | --- | --- |
| 1 | Question/objective sufficiently described? | 2 | 2 | 2 | 2 | 2 | 2 | 2 | 2 | 2 | 2 | 2 |
| 2 | Study design evident and appropriate? | 2 | 2 | 2 | 2 | 2 | 2 | 2 | 2 | 2 | 2 | 2 |
| 3 | Method of subject/comparison group selection or source of information/input variables described and appropriate? | 2 | 2 | 1 | 2 | 2 | 2 | 2 | 2 | 2 | 2 | 1 |
| 4 | Subject (and comparison group, if applicable) characteristics sufficiently described? | 2 | 1 | 2 | 1 | 2 | 2 | 2 | 2 | 2 | 2 | 2 |
| 5 | If interventional and random allocation was possible, was it described? | N/A | N/A | N/A | N/A | N/A | N/A | N/A | N/A | N/A | N/A | N/A |
| 6 | If interventional and blinding of investigators was possible, was it reported? | N/A | N/A | N/A | N/A | N/A | N/A | N/A | N/A | N/A | N/A | N/A |
| 7 | If interventional and blinding of subjects was possible, was it reported? | N/A | N/A | N/A | N/A | N/A | N/A | N/A | N/A | N/A | N/A | N/A |
| 8 | Outcome and (if applicable) exposure measure(s) well defined and robust to measurement/misclassification bias? Means of assessment reported? | 2 | 2 | 2 | 2 | 2 | 2 | 2 | 2 | 2 | 2 | 2 |
| 9 | Sample size appropriate? | 1 | 1 | N/A | 1 | 1 | 1 | 1 | N/A | 1 | 1 | N/A |
| 10 | Analytic methods described/justified and appropriate? | 2 | 1 | 2 | 2 | 2 | 2 | 2 | 2 | 2 | 2 | 2 |
| 11 | Some estimate of variance is reported for the main results? | 2 | 2 | 2 | 2 | 0 | 1 | 2 | 2 | 2 | 2 | 2 |
| 12 | Controlled for confounding? | 0 | 2 | 2 | 1 | 1 | 2 | 2 | 2 | 2 | 2 | 2 |
| 13 | Results reported in sufficient detail? | 2 | 2 | 2 | 2 | 1 | 2 | 2 | 2 | 2 | 2 | 2 |
| 14 | Conclusions supported by the results? | 2 | 2 | 2 | 2 | 2 | 2 | 1 | 2 | 2 | 2 | 2 |
|  | **Total sum** | **19** | **19** | **19** | **19** | **17** | **20** | **20** | **20** | **21** | **21** | **19** |
|  | **Total possible sum** | **22** | **22** | **20** | **22** | **22** | **22** | **22** | **20** | **22** | **22** | **20** |
|  | **Summary Score (total sum / total possible sum)** | **0.86** | **0.86** | **0.95** | **0.86** | **0.77** | **0.91** | **0.91** | **1.00** | **0.95** | **0.95** | **0.95** |

| Criteria | QUESTION | Stavropoulos et al. (2020) | Stavropoulos et al. (2021) | Stockdale & Coyne (2020) | Sung, Kim, & Cho (2020) | Throuvala, Janikian, Griffiths, Rennoldson, & Kuss (2019) | Tullett-Prado, Stavropoulos, Mueller, Sharples, & Footitt (2021) | | Wang et al. (2014) | | Wartberg, Kriston, & Kammerl (2017) |
| --- | --- | --- | --- | --- | --- | --- | --- | --- | --- | --- | --- |
| 1 | Question/objective sufficiently described? | 2 | 2 | 2 | 2 | 2 | 2 | 1 | | 2 | |
| 2 | Study design evident and appropriate? | 2 | 2 | 2 | 2 | 2 | 2 | 2 | | 2 | |
| 3 | Method of subject/comparison group selection or source of information/input variables described and appropriate? | 1 | 1 | 2 | 2 | 2 | 1 | 2 | | 2 | |
| 4 | Subject (and comparison group, if applicable) characteristics sufficiently described? | 2 | 2 | 2 | 2 | 1 | 2 | 2 | | 2 | |
| 5 | If interventional and random allocation was possible, was it described? | N/A | N/A | N/A | N/A | N/A | N/A | N/A | | N/A | |
| 6 | If interventional and blinding of investigators was possible, was it reported? | N/A | N/A | N/A | N/A | N/A | N/A | N/A | | N/A | |
| 7 | If interventional and blinding of subjects was possible, was it reported? | N/A | N/A | N/A | N/A | N/A | N/A | N/A | | N/A | |
| 8 | Outcome and (if applicable) exposure measure(s) well defined and robust to measurement/misclassification bias? Means of assessment reported? | 2 | 2 | 2 | 2 | 2 | 2 | 2 | | 2 | |
| 9 | Sample size appropriate? | N/A | 1 | 1 | 1 | N/A | 0 | N/A | | N/A | |
| 10 | Analytic methods described/justified and appropriate? | 2 | 2 | 2 | 2 | 2 | 2 | 2 | | 2 | |
| 11 | Some estimate of variance is reported for the main results? | 2 | 2 | 2 | 2 | 2 | 2 | 2 | | 2 | |
| 12 | Controlled for confounding? | 2 | 2 | 0 | 0 | 0 | 2 | N/A | | 2 | |
| 13 | Results reported in sufficient detail? | 2 | 2 | 2 | 2 | 2 | 2 | 2 | | 2 | |
| 14 | Conclusions supported by the results? | 2 | 2 | 2 | 2 | 2 | 2 | 2 | | 2 | |
|  | **Total sum** | **19** | **20** | **19** | **19** | **17** | **19** | **17** | | **20** | |
|  | **Total possible sum** | **20** | **22** | **22** | **22** | **20** | **22** | **18** | | **20** | |
|  | **Summary Score (total sum / total possible sum)** | **0.95** | **0.91** | **0.86** | **0.86** | **0.85** | **0.86** | **0.94** | | **1.00** | |

**Appendix B. Descriptions for five diagnostic tools**

*1. Internet Gaming Disorder Scale-Short Form (IGDS9-SF)*

IGDS9-SF consists of nine items that assess the severity of IGD symptoms (Pontes & Griffiths, 2015). The scale was developed based on the DSM-5 criteria for IGD (American Psychiatric Association, 2013). All items are rated on a 5-point Likert scale, ranging from 1 (never) to 5 (very often). Individual item scores are summed and averaged to create a composite score, with higher scores suggesting greater IGD severity.

*2. Seven-Item Game Addiction Scale (GAS-7)*

GAS-7 is the short version of the GAS measuring gaming disorder in adolescents (Lemmens, Valkenburg, & Peter, 2009). Seven items are based on seven DSM-IV criteria for pathological gambling including salience, tolerance, mood modification, withdrawal, relapse, conflict, and problems (American Psychiatric Association, 1994). Items are rated on a 5-point Likert scale, ranging from 1 (never) to 5 (very often). Four items scoring higher than 2 (sometimes or more) indicates problematic use of video games. In the original paper, the GAS-7 had a Cronbach's alpha of .86 in the first sample and .81 in the second sample (Lemmens et al., 2009).

*3. Internet Gaming Disorder Scale (Lemmens IGD-9)*

The Internet Gaming Disorder Scale (IGDS) has four versions: a long (27-items) and short (9-items) polytomous scale and a long (27-items) and short (9-items) dichotomous scale. Most of the studies included in this review used the dichotomous 9-item version, which comprises nine items on the presence of IGD symptoms based on the nine criteria from the DSM-5 (Lemmens, Valkenburg, & Gentile, 2015). All items are rated as a binary response (no = 0, yes = 1) and the total score is calculated by summing up the responses. Participants who respond yes to five or more items are classified as having IGD and higher scores indicate more severe cases of IGD. The dichotomous 9-item IGDS showed good reliability with a Cronbach’s alpha of .83.

*4. Assessment of Internet and Computer Addiction Scale-Gaming (AICA-Sgaming)*

AICA-S is a 14-item self-report assessing IGD based on the DSM-IV criteria for gambling and substance-related disorders (Klaus Wölfling, Beutel, & Müller, 2012). Fourteen items cover clinical classification relevant for internet use behavior (e.g., craving, tolerance, withdrawal, loss of control, and unsuccessful attempts to cut back), negative repercussions of internet use according to six areas (e.g., problems with school, work etc.), and time spent online and preferred online activity. A cutoff score of 7 (3 criteria fulfilled) indicates a moderate addictive use and a score of 13.5 (5 criteria fulfilled) or higher is considered a severe addictive use. This scale had good reliability (internal consistency of ɑ = .89) and validity (factorial and construct validity) (Müller & Wölfling, 2010; K. Wölfling, Müller, & Beutel, 2011).

*5. Ten-Item Internet Gaming Disorder Test (IGDT-10)*

IGDT-10 comprises 10 items that assess IGD based on the DSM-5 criteria of IGD (Király et al., 2017). All items are rated on a 3-point Likert scale (never, sometimes, often) but scoring is dichotomized (“never” / “sometimes” = 0, “often” = 1). The composite score ranges from 0 to 9; items 9 and 10 belong to the same criterion thus only a single score is used. The cut-off threshold, as proposed by DSM-5, is the endorsement of five or more criteria and higher scores indicate more severe cases of IGD. The original paper reported Cronbach's alpha of .68 and a Guttman's Lambda-2 value of .69.

**REFERENCES FOR SUPPLEMENTARY MATERIALS**

American Psychiatric Association, A. (1994). *Diagnostic and statistical manual of mental disorders* (Vol. 4th ed): Washington, DC: American psychiatric association.

American Psychiatric Association, A. (2013). *Diagnostic and statistical manual of mental disorders: DSM-5* (Vol. 10): Washington, DC: American psychiatric association.

Király, O., Sleczka, P., Pontes, H. M., Urbán, R., Griffiths, M. D., & Demetrovics, Z. (2017). Validation of the ten-item Internet Gaming Disorder Test (IGDT-10) and evaluation of the nine DSM-5 Internet Gaming Disorder criteria. *Addictive Behaviors, 64*, 253-260. doi:10.1016/j.addbeh.2015.11.005

Lemmens, J. S., Valkenburg, P. M., & Gentile, D. A. (2015). The Internet gaming disorder scale. *Psychological assessment, 27*(2), 567. doi:10.1037/pas0000062

Lemmens, J. S., Valkenburg, P. M., & Peter, J. (2009). Development and validation of a game addiction scale for adolescents. *Media psychology, 12*(1), 77-95. doi:10.1080/15213260802669458

Müller, K. W., & Wölfling, K. (2010). Pathological computer game and internet use: Scientific insights into phenomenology, epidemiology, diagnosis and comorbidity. *Suchtmedizin in Forschung und Praxis, 12*, 45-55.

Pontes, H. M., & Griffiths, M. D. (2015). Measuring DSM-5 Internet gaming disorder: Development and validation of a short psychometric scale. *Computers in human behavior, 45*, 137-143. doi:10.1016/j.chb.2014.12.006

Wölfling, K., Beutel, M., & Müller, K. (2012). Construction of a Standardized Clinical Interview to Assess Internet addiction: First Findings Regarding the Usefulness of AICA-C. *Addiction Research & Therapy, 6*, 1-7. doi:10.4172/2155-6105.S6-003

Wölfling, K., Müller, K. W., & Beutel, M. (2011). [Reliability and validity of the Scale for the Assessment of Pathological Computer-Gaming (CSV-S)]. *Psychother Psychosom Med Psychol, 61*(5), 216-224. doi:10.1055/s-0030-1263145
